# Critical Role of Nrf2 in Experimental Ischemic Stroke

**DOI:** 10.3389/fphar.2019.00153

**Published:** 2019-03-05

**Authors:** Lei Liu, Logan M. Locascio, Sylvain Doré

**Affiliations:** ^1^Department of Anesthesiology, Center for Translational Research in Neurodegenerative Disease and McKnight Brain Institute, University of Florida, Gainesville, FL, United States; ^2^Departments of Neurology, Psychiatry, Pharmaceutics, and Neuroscience, University of Florida, Gainesville, FL, United States

**Keywords:** antioxidant response element, global cerebral ischemia, middle cerebral artery occlusion, permanent MCAO, preclinical models, reperfusion, stroke, transient MCAO

## Abstract

Ischemic stroke is one of the leading causes of death and long-term disability worldwide; however, effective clinical approaches are still limited. The transcriptional factor Nrf2 is a master regulator in cellular and organismal defense against endogenous and exogenous stressors by coordinating basal and stress-inducible activation of multiple cytoprotective genes. The Nrf2 network not only tightly controls redox homeostasis but also regulates multiple intermediary metabolic processes. Therefore, targeting Nrf2 has emerged as an attractive therapeutic strategy for the prevention and treatment of CNS diseases including stroke. Here, the current understanding of the Nrf2 regulatory network is critically examined to present evidence for the contribution of Nrf2 pathway in rodent ischemic stroke models. This review outlines the literature for Nrf2 studies in preclinical stroke and focuses on the *in vivo* evidence for the role of Nrf2 in primary and secondary brain injuries. The dynamic change and functional importance of Nrf2 signaling, as well as Nrf2 targeted intervention, are revealed in permanent, transient, and global cerebral ischemia models. In addition, key considerations, pitfalls, and future potentials for Nrf2 studies in preclinical stroke investigation are discussed.

## Introduction

Ischemic stroke is one of the leading causes of death and long-term disability worldwide (Benjamin et al., 2018), an event which is more disabling than it is fatal. In the U.S. alone, ~795,000 people each year suffer a new or recurrent stroke, of which 87% are ischemic (Dirnagl et al., 2003; Feigin et al., 2017; Benjamin et al., 2018). However, effective clinical approaches are still limited. A successful therapeutic strategy for salvaging ischemic brain tissue and promoting functional outcomes is to quickly restore blood flow during acute ischemic stroke by introducing intravenous recombinant tissue plasminogen activator (rtPA), which has been available since 1996 (Prabhakaran et al., 2015; Romano and Sacco, 2015), in combination with thrombectomy. However, more than 95% of patients do not benefit from rtPA due to the narrow therapeutic window (4.5 h) and limited indications (Hacke et al., 2008; Fonarow et al., 2011; Sandercock et al., 2012; Emberson et al., 2014). In addition, there is no efficacious treatment that exhibits long-term recovery improvement. Therefore, development of new therapeutic strategies by targeting vital cellular components of the ischemic cascade is urgently needed.

The transcriptional factor Nrf2 is a major regulator of cellular and organismal defense mechanism against endogenous and exogenous stresses by coordinating basal and stress-inducible activation of multiple cytoprotective genes (Leonardo and Doré, 2011; Suzuki et al., 2013; Hayes and Dinkova-Kostova, 2014; Tonelli et al., 2017; Cuadrado et al., 2018; Yamamoto et al., 2018). Since its discovery in 1994 (Moi et al., 1994), Nrf2 biology has been extensively studied in understanding the structure, molecular mechanism, function, regulation of Nrf2 activity, downstream pathways and implications as a therapeutic target of diseases (Ma, 2013; Suzuki et al., 2013; Kumar et al., 2014; Cuadrado et al., 2018; Yamamoto et al., 2018). It is now recognized as a master regulator of redox homeostasis through the control of a wide array of target genes that share a common DNA sequence called the antioxidant response element (ARE) in the promoter region ensuring that its activity increases in response to redox perturbation, energy or nutrient fluxes, inflammation, toxicity, and disease conditions (Ma, 2013; Cuadrado et al., 2018; Yamamoto et al., 2018). The equivalent response element in mice and rats is called the electrophile response element (EpRE) (Friling et al., 1990; Wasserman and Fahl, 1997; Itoh et al., 2010; Yamamoto et al., 2018). Besides mediating antioxidant responses, Nrf2 contributes to the regulation of many primary and secondary metabolic processes (Hayes and Dinkova-Kostova, 2014; Tonelli et al., 2017). Consequently, targeting Nrf2 has emerged as an attractive therapeutic strategy for stroke prevention and reversal (Calkins et al., 2009; Leonardo and Doré, 2011; Wang Y. C. et al., 2011; Ma, 2013; Kumar et al., 2014; Tonelli et al., 2017).

This paper outlines the current understanding of the Nrf2 regulatory network and critically examined the recent evidence for the contribution of Nrf2 pathway in experimental ischemic stroke models. It includes extensive literature review for Nrf2 studies in experimental ischemic stroke mouse and rat models published by June 30, 2018 with a special focus on *in vivo* evidence for the role of Nrf2 in ischemic brain injury. We summarized the dynamic regulation of Nrf2 signaling, functional importance, and its targeted intervention in permanent, transient, and global cerebral ischemia preclinical models. Finally, we also assessed key considerations, pitfalls, and the potential for future Nrf2 studies in stroke investigation.

## Overview of the Nrf2 Regulatory Network

### A Brief History

Nrf2 is widely expressed in mammalian cells. In 1991, a study in the field of toxicology revealed that oxidative stress activates antioxidant genes through the antioxidant-response element (ARE), a cis-acting regulatory element that contributes to cellular defense in eukaryotes (Rushmore et al., 1991; Hayes and Dinkova-Kostova, 2014). In 1994, Nrf2 was firstly reported in the molecular biology field as an NF-E2-like basic leucine zipper transcriptional activator that binds to the tandem NF-E2/AP1 repeat of the beta-globin locus control region (Moi et al., 1994; Raghunath et al., 2018). However, its biological function was unclear. In 1996, the first study on Nrf2 knockout mice showed that no overt abnormal phenotype was detected although the mice were susceptible to stresses (Chan et al., 1996). In 1997, a landmark study illustrated that the induction of two ARE-driven genes, glutathione S-transferase (GST) and NAD(P)H:quinone oxidoreductase-1 (NQO1), was abolished in the Nrf2 knockout mice by the phenolic antioxidant butylated hydroxyanisole (BHA), revealing that Nrf2 controls drug-metabolizing enzymes *in vivo* (Itoh et al., 1997). Recently, series of breakthrough studies have revealed that Nrf2 coordinately regulates a wide array of antioxidant response element/electrophile responsive element (ARE/EpRE)-driven genes that play critical roles in controlling endogenous resistance to various intrinsic and extrinsic stressors (Ma, 2013; Suzuki et al., 2013; Hayes and Dinkova-Kostova, 2014; Tonelli et al., 2017; Yamamoto et al., 2018).

### Structure Domain of Nrf2

**Figure 1:**
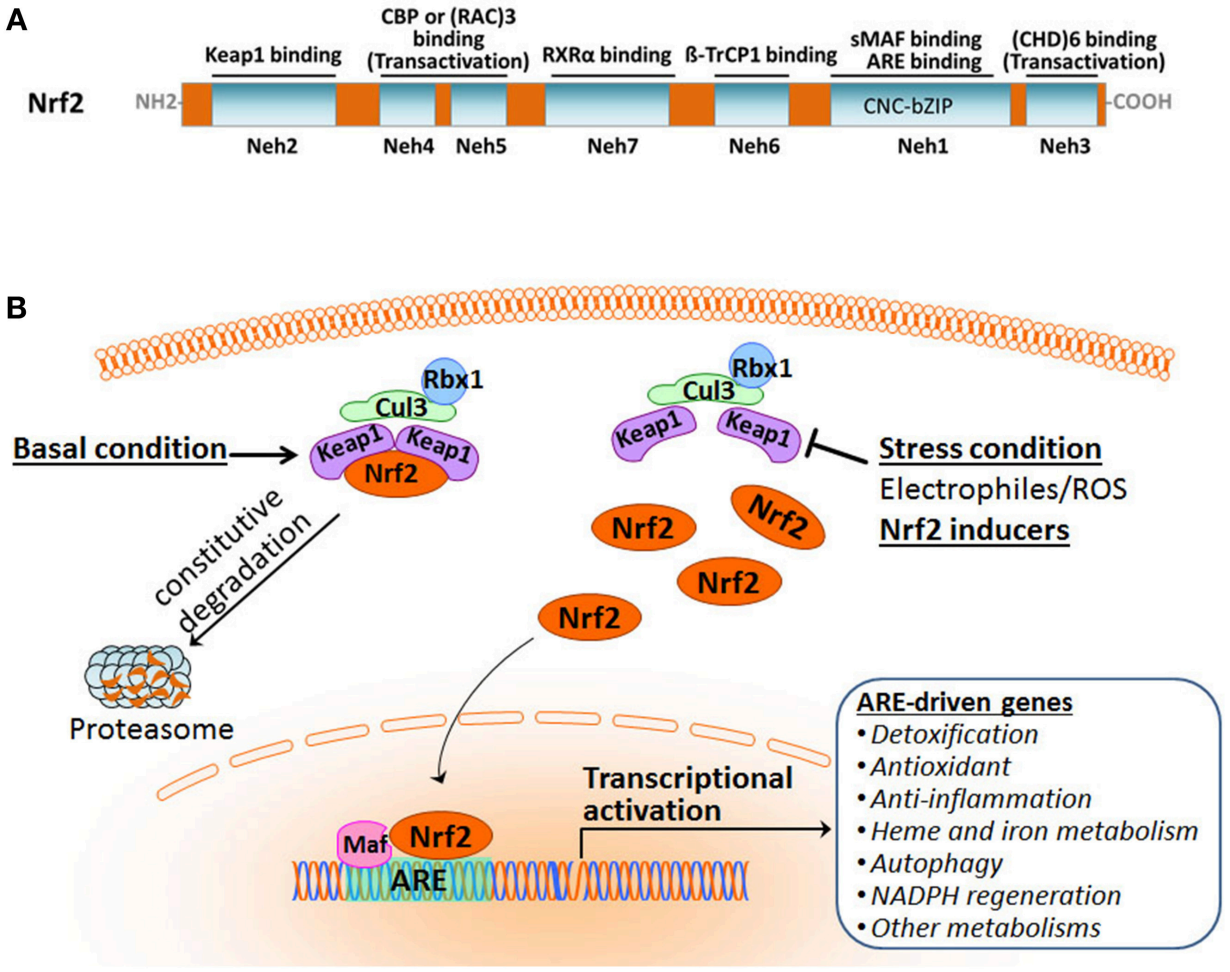
Overview of the Nrf2 pathway activation. **(A)** Functional domains of human Nrf2 protein. **(B)** The Keap1-dependent Nrf2 activation and response.

Nrf2 belongs to the cap'n'collar subfamily of the basic-region leucine zipper (CNC-bZIP) transcription factors. It is a modular protein composed of seven functional domains, Nrf2-ECH homology (Neh) domains 1–7, which have distinct functions (Figure 1A) (Chan et al., 1993; Katoh et al., 2005; Hayes and Dinkova-Kostova, 2014; Tonelli et al., 2017). The Neh1 domain contains the well-conserved CNC-bZIP region that heterodimerizes with small musculoaponeurotic fibrosarcoma oncogene (sMaf) proteins and binds ARE/EpRE sequence in DNA. This CNC-bZIP region, which can be found in several species, is vital for Nrf2 function. The N-terminal Neh2 is the domain by which Nrf2 binds to its primary negative regulator Keap1 through its low-affinity DLG and the high-affinity ETGE motifs. The C-terminal Neh3 region represents a transactivation domain that binds to chromo-ATPase/ helicase DNA-binding protein (CHD) 6 and activates transcription of Nrf2 target genes in concert with the Neh4 and Neh5 domains. The Neh4 and Neh5 regions of Nrf2 are transactivation domains that recruit the cAMP response element-binding protein (CREB)-binding protein (CBP) and/or receptor-associated coactivator (RAC) 3. The Neh6 domain, independent of Keap1, mediates the repression of Nrf2 stability with another negative regulator the dimeric β-transducin repeat-containing protein (β-TrCP), a substrate adaptor for the S-phase kinase-associated protein 1 (Skp1)–Cul1–Rbx1 core E3 complex, through DSGIS and DSAPGS motifs. The Neh7 domain is involved in the negative control of Nrf2 by physically binding to the retinoid X receptor (RXR).

### Overview of the Nrf2 Pathway

Organisms are equipped with a defense system to maintain homeostasis against constant intrinsic and extrinsic insults that result in the damage of nucleic acids, proteins, and membrane lipids. Nrf2 is a master regulator of the inducible cell defense system by controlling a broad range of cytoprotective genes (Hayes and Dinkova-Kostova, 2014; Suzuki and Yamamoto, 2015). Under basal homeostatic conditions, Nrf2 in the cytoplasm is predominately bound to the Kelch-like ECH-associating protein 1 (Keap1) through the Keap1–Cullin3 (Cul3)-Rbx1 ubiquitin E3 ligase complex and is constitutively degraded by the proteasome. Cul3 serves as a scaffolding protein that is bound to both Keap1 and Rbx1. As a result, Nrf2 abundance and activity are maintained at low levels (Figure 1B). Under stress conditions, Nrf2 protein is liberated from Keap1-mediated repression. The stabilized and accumulated Nrf2 translocates into the nucleus and, as a heterodimer with one of the small Maf proteins, binds to the ARE/EpRE in the promoter region of Nrf2 target genes, thus activating a wide array of cytoprotective genes (Ma, 2013; Kumar et al., 2014; Suzuki and Yamamoto, 2015; Taguchi and Yamamoto, 2017;Bellezza et al., 2018; Yamamoto et al., 2018).

It is becoming increasingly clear that Keap1 plays a central role in the regulatory mechanism of Nrf2. (1) Keap1 acts as a sensor of oxidative and electrophilic stresses for Nrf2 with a subcellular localization in the perinuclear cytoplasm (Suzuki and Yamamoto, 2017; Yamamoto et al., 2018). (2) Keap1 is a major repressor of Nrf2 through the activity of the Keap1–Cul3 complex. It is an adaptor protein for Cul3-dependent ubiquitination and specifically targets Nrf2 (Itoh et al., 1999, 2003; Kobayashi et al., 2004), thereby marking Nrf2 protein for rapid degradation by the ubiquitin–proteasome system. Under normal conditions, Nrf2 undergoes constitutive ubiquitination by the Keap1-Cul3 complex and resultant proteasomal degradation, leading to its low level of activity. Exposure to cellular insults like electrophiles or reactive oxygen species (ROS) brings activity of the E3 ubiquitin ligase Keap1-Cul3 complex to a halt, inhibiting Nrf2 ubiquitination and enabling Nrf2 protein stabilization and transcriptional activation. (3) Keap1 serves as a “floodgate” for Nrf2: Normally it functions to suppress Nrf2 nuclear translocation in response to cellular insults, the gene promotes translocation of Nrf2 into the nucleus to activate cytoprotective target gene expression. Keap1 has a relatively long half-life of 12 h compared to Nrf2's half-life of 20 min, contributing to its importance in the Nrf2 regulatory network.

### Target Genes and Functions of Nrf2

The Nrf2-Maf complex binds to the specific promoter of its target genes. In the past decade, more than 200 target genes of Nrf2 have been identified through the analyses of gene expression profiling and chromatin immunoprecipitation (ChIP) (Suzuki et al., 2013; Kumar et al., 2014; Suzuki and Yamamoto, 2017; Rojo de la Vega et al., 2018). The Nrf2 target ARE-driven genes have been defined in encoding proteins for functional regulation of a wide range of biological processes including detoxification, antioxidation, anti-inflammation, NADPH regeneration, and intermediary metabolisms (Figure 1B). Nrf2 exerts multiple defense processes counteracting various stresses by the induction of these genes. Consequently, Nrf2 plays a fundamental role in maintaining the redox homeostasis of the cell.

### Target Genes and Functions of Nrf2—*Detoxification of Xenobiotics*

Xenobiotics, including toxic chemicals and drugs, are detoxified by various drug-metabolizing enzymes and transporters. This detoxification mechanism is divided into three phases (Croom, 2012; Raghunath et al., 2018). Phase I enzymes mainly consist of cytochrome P450 oxidases that modify the xenobiotics through oxidation, reduction, or hydrolysis. Phase II conjugating enzymes include glutathione S-transferases, which catalyze the conjugation of reactive electrophile species with glutathione (GSH), attenuating the toxic potential of xenobiotics. Phase III transporters eliminate the GSH conjugates and offer protection against deleterious chemicals. It has been demonstrated that Nrf2 controls the expression of many drug-metabolizing enzymes [like distinct GST subunits and [NAD(P)H:quinone oxidoreductase 1) NQO1] and transporters [(drug-resistance-associated proteins (MRP)].

### Target Genes and Functions of Nrf2—*Antioxidant*

A major function of Nrf2 is to resist oxidative stress, thereby maintaining redox homeostasis between reactive oxidants and endogenous antioxidant systems. Groups of Nrf2 targeted genes that regulate antioxidant defense and oxidant signaling have been defined. (1) Glutathione (GSH)-based: glutamate–cysteine ligase catalytic (GCLC) subunit and glutamate-cysteine ligase modifier (GCLM) control the entry of cystine into cells; glutathione peroxidase (GPX) 2 produces oxidized glutathione (GSSG) during the reduction of peroxides; and glutathione reductase (GSR) 1 reduces GSSG for maintenance of reduced intracellular GSH levels. (2) Thioredoxin (TXN)-based: Thioredoxin (TXN) 1, thioredoxin reductase (TXNRD) 1, and sulfiredoxin (SRXN) 1 reduce oxidized protein thiols. (3) Others: G6PDH and 6PGD reduce synthesis of NADPH, antioxidant protein Trx, stress response protein heme oxygenase 1 (HO1), and other proteins. Nrf2 coordinately regulates key components in the antioxidant system that precisely controls antioxidant defense at multiple levels, thus ensuring an adequate response to oxidants in time and space (Lin and Beal, 2006; Ma, 2013).

### Target Genes and Functions of Nrf2—*Anti-inflammation*

Besides protecting against xenobiotic and oxidative insults, Nrf2 plays a role in anti-inflammation, which is supported by several recent findings (Kobayashi et al., 2016; Suzuki and Yamamoto, 2017). Several findings support the anti-inflammatory effect. The absence of Nrf2 leads to exacerbated inflammation in different murine models. Attenuated inflammation by Nrf2 is related to the inhibition of the nuclear factor-κB (NF-κB) pathway and proinflammatory cytokine level (Wardyn et al., 2015). Most recently, it was demonstrated that Nrf2 suppressed lipopolysaccharide (LPS)-induced inflammation by blocking the transcriptional upregulation of proinflammatory cytokines, including IL-6 and IL-1β. ChIP-seq and ChIP-qPCR analyses revealed that Nrf2 binds near the promoter region of these genes, and this Nrf2-mediated inhibition is independent of the Nrf2-binding motif and ROS level (Ahmed et al., 2017).

### Target Genes and Functions of Nrf2—*Heme and Iron Metabolism*

Nrf2 also modulates the key components of heme and iron metabolism. HO1 catalyzes the cleavage of heme (which cannot be recycled) to form iron, carbon monoxide (CO), and biliverdin, which is immediately reduced to bilirubin (Doré and Snyder, 1999; Doré et al., 1999). HO1 is considered to be a stress protein that can be highly induced in a cell-specific manner by numberous conditions (Ewing et al., 1992). Iron is intimately linked to oxygen by acting as an essential nutrient cofactor in enzymes for oxygen transport, oxidative phosphorylation, and metabolite oxidation. However, excess labile iron—not bound to ferritin—would facilitate the formation of oxygen-derived free radicals capable of damaging biomolecules (Rouault, 2013; Kerins and Ooi, 2017). The biological utilization of iron is a tightly regulated process. Constitutive Nrf2 activation and subsequent deregulation of iron metabolism have been implicated in cancer development. Nrf2-mediated upregulation of HO1 or the iron storage protein ferritin can lead to enhanced proliferation and therapy resistance.

### Target Genes and Functions of Nrf2—NADPH Regeneration and Other Metabolic Processes

NADPH is an essential cofactor for many drug-metabolizing enzymes and antioxidants. Nrf2 controls NADPH production by regulating key NADPH-generating enzymes, such as glucose-6-phosphate dehydrogenase (G6pd), 6-phosphogluconate dehydrogenase (Pgd), isocitrate dehydrogenase 1 (Idh1), and malic enzyme 1 (Leonardo and Doré, 2011; Dinkova-Kostova and Abramov, 2015). In addition, Nrf2 controls the expression of multiple metabolic enzymes, indicating its unique role between redox and intermediary metabolism. Nrf2 also regulates fatty acid oxidation and lipases that influence lipid metabolism and transcription factors (Hayes and Dinkova-Kostova, 2014).

### Regulatory Mechanisms of Nrf2

Besides the predominant view that Nrf2 activity is mainly regulated at the protein stability level by Keap-mediated repression, accumulated evidence show that Nrf2 activity is tightly controlled at the transcriptional and post-translational levels during both basal and stressful conditions (Hayes and Dinkova-Kostova, 2014; Tonelli et al., 2017; Yamamoto et al., 2018). Given the central role of Nrf2 in cellular defense, the regulatory mechanisms of Nrf2 activity are intricate and multifaceted.

### Regulatory Mechanisms of Nrf2–Transcriptional Regulation

Nrf2 mRNA is broadly expressed across different species (Ma, 2013; Tonelli et al., 2017; Yamamoto et al., 2018), and its basal levels vary within different organs. Nrf2 activity is at least partially regulated at the transcriptional level. (1) The rodent Nrf2 gene comprises two ARE-like sequences in its promoter region and, in response to electrophilic stimuli, appropriately autoregulates its own expression level in concert with sustaining the induction of ARE-driven genes, offering a positive feedback mechanism to augment the Nrf2 signal. In contrast, the ARE-like sequences in the human Nrf2 gene promoter region are reportedly associated with reduced expression of Nrf2, which enhances vulnerability to lung cancer. (2) The Nrf2 gene promoter comprises a binding site for NF-κB, which can be induced by environmental stress, injury or inflammation. Indeed, Nfe2l2 transcription level in human monocytes is indeed activated by LPS-induced inflammation. (3) It was reported that Nfe2l2 transcription in tumor cells can be amplified by the Notch signaling pathway and the phosphoinositide 3-kinase (PI3K)-Akt pathway, ultimately contributing to the increased expression of Nrf2.

### Regulatory Mechanisms of Nrf2—Post-transcriptional Regulation

A microRNA (miRNA) is a small non-coding RNA molecule that functions in RNA silencing and post-transcriptional regulation of gene expression. At least eight miRNAs have been identified as direct modulators of Nrf2 expression at the transcriptomic level (Ayers et al., 2015). The miR-144 was identified to negatively regulate Nrf2 level in reticulocytes of homozygous sickle cell disease (HbSS) patients (Bryan et al., 2013; Kurinna and Werner, 2015) (140). Increased miR-144 is associated with the reduction of both Nrf2 level and GSH regeneration as well as impeded oxidative stress defense.

### Regulatory Mechanisms of Nrf2—Post-translational Regulation in Nrf2 Protein Stability

#### Keap1-Dependent Repression Mechanism

(1) The prevailing view is that Keap1 is a primary repressor of Nrf2. Under basal conditions, Keap1 constantly targets Nrf2 for ubiquitination and proteasomal degradation, resulting in the disruption of Nrf2 protein stability and maintenance of Nrf2 signaling capacity at a very low level. In response to electrophiles or stressors, Nrf2 is liberated from the Keap1 repression (i.e., derepression) by inhibiting ubiquitylation and proteasomal degradation that allows newly-synthesized Nrf2 to be rapidly stabilized and activates transcriptional activation of cytoprotective genes. This view is supported by many reports of Keap1 knockout mice and Keap1 knockdown human cells that exhibited sufficient Nrf2 activity (Kensler et al., 2007; Yates et al., 2009; Bellezza et al., 2018; Yamamoto et al., 2018). Blockage of Keap1 expression by miR-141 or miR-200a triggers increased Nrf2 activity at the protein stabilization level. Transgenic complementation rescue assay is a comprehensive and powerful approach to delineate the *in vivo* functions of proteins (Motohashi et al., 2011; Katsuoka and Yamamoto, 2016). The esophagus lesions and resultant mortality in Keap1 knockout mice were fully retrieved by concurrent Nrf2 disruption, and aberrant phenotypes caused by Keap1 deficiency can also be rescued by transgene-derived Keap1. Epigenetic silencing of the Keap1 gene by hypermethylation of its promoter results in upregulation of Nrf2 in patients with gliomas and different cancers of breast, lung, prostate and colorectal (Tonelli et al., 2017). (2) The function of Keap1 within various electrophiles is related primarily to three critical cysteine residues: Cys151, Cys273, and Cys288 (Yamamoto et al., 2018). Keap1 is a thiol-rich protein with many cysteine residues and thus is sensitive to electrophiles (McMahon et al., 2010). Therefore, covalent modifications of the cysteine residues had been proposed to influence the function of Keap1, which releases Nrf2 (McMahon et al., 2010). Affirmatively, covalent binding of electrophiles to critical cysteine residues has been observed. Meanwhile, various lines of studies in cultured cells, zebrafish, and mice demonstrate that Keap1 functions as a sensor due to the prominent contribution of the cysteine residues. Multiple studies involving substitution of Cys151 strongly support its role as a main cysteine sensor by modulating the activity of the ubiquitin E3 ligase Keap1-Cul3 complex to respond to different electrophiles. (3) To activate Nrf2 by inhibiting Keap1 activity: Many ARE activity inducing agents are soft electrophiles that inhibit Keap1 by modifying Cys151, Cys226/Cys613, Cys-273/Cys-288, or Cys-434. Thus, the presence of multiple Cys-based sensors in Keap1 allows Nrf2 to be de-repressed in response to many xenobiotic stressors. (4) To activate Nrf2 by competitive inhibition of Keap1: p62, the first mammalian selective autophagy cargo receptor (Chu, 2018), is able to directly interact with the Nrf2- binding site on Keap1, a component of Cullin-3-type ubiquitin ligase for Nrf2 (Komatsu et al., 2010; Lau et al., 2010). Thus, an overproduction of p62 or a deficiency in autophagy competes with the interaction between Nrf2 and Keap1, resulting in Nrf2 stabilization and transcriptional activation of Nrf2 target genes. Additionally, p62 is a target gene of Nrf2 and creates a positive feedback loop by inducing ARE-driven gene transcription (Jain et al., 2010). Oxidative stress-triggered Nrf2 results in the induction and accumulation of p62 that in turn activates Nrf2 activation (Komatsu et al., 2010).

#### Keap1-Nondependent Repression Mechanism

(1) The Nrf2 is has been described to be suppressed by b-TrCP and GSK-3; in addition to the well-studied Keap1, E3 ubiquitin ligase adaptor β-TrCP is another negative repressor of Nrf2 stabilization (Rada et al., 2012). Nrf2 is controlled by two distinct β-TrCP recognition motifs in its Neh6 domain, one of which can be modulated by GSK-3 activity (Chowdhry et al., 2013). Intraperitoneal injection of the GSK-3 inhibitor through the GSK-3/β-TrCP axis led to increased Nrf2 and HO1 levels in liver and hippocampus (Rada et al., 2012). Activation of the GSK3β/β-TrCP axis by gene knockdown of PHLPP1 in NRK52E cells enhanced Nrf2-responsive antioxidant enzymes HO1 and NQO1 (Mathur et al., 2018). (2) The Nrf2 is suppressed by CRIF1 and RNF4: CR6-interacting factor 1(CRIF1) physically interacts with both N- and C-terminal regions of Nrf2 and promotes Nrf2 ubiquitination and subsequent proteasome-mediated Nrf2 protein degradation (Kang et al., 2010). *Crif1*-knockdown BMMSCs caused increased oxidative stress and apoptosis after irradiation injury, partially due to a suppressed antioxidant response mediated by decreased Nrf2 nuclear translocation (Chen L. et al., 2017). RING finger protein 4 (Rnf4) mediates polyubiquitylation of polysumoylated Nrf2, leading to its subsequent degradation in promyelocytic leukemia-nuclear bodies (Malloy et al., 2013). Respiratory syncytial virus-induced Nrf2 degradation occurs in a SUMO-specific E3 ubiquitin ligase—RING finger protein 4 (Rnf4)-dependent manner.

## Cerebral Ischemia And Relevant Preclinical Animal Models

Ischemic stroke is caused by cerebral arterial occlusion that leads to a critical reduction or loss of regional cerebral blood flow, and it occurs as a consequence of multiple vascular diseases, including cardioembolism, atherosclerosis, small vessel disease, and cryptogenic diseases (Adams and Biller, 2015; Mehndiratta et al., 2015). Interruption of the blood supply initiates complex spatial and temporal events involving hemodynamic, biochemical, and neurophysiologic alterations that ultimately lead to a pathological disturbance and a wide range of clinical symptoms (Lo et al., 2003; Iadecola and Anrather, 2011). The severity and temporal evolution of ischemic injury depend on the extent of cerebral blood flow (CBF), localization, duration of ischemia and coexisting systemic diseases among individual patients (Martini and Kent, 2007; Bang et al., 2008; Shen and Duong, 2008; Liebeskind, 2010).

Experimental ischemic stroke models are indispensable to our understanding of the events occurring in the ischemic and reperfused brains, enabling us to elucidate the pathophysiological mechanisms of ischemic brain injury and develop novel therapeutic treatments (Bosetti et al., 2017; Quinn et al., 2018). The vast majority of stroke models are carried in rats or mice because they present clear advantages of lower cost, resemblance to human cerebrovascular anatomy and physiology, and reproducibility of studies (Durukan and Tatlisumak, 2007; Durukan et al., 2008). In addition, genetic modification is usually applied in mice to illuminate molecular pathophysiological conditions like stroke. Cerebral ischemia in humans is divided into two categories: focal and global. Focal cerebral ischemia occurs when CBF is disrupted within a specific brain region, whereas global cerebral ischemia occurs when CBF is blocked throughout most or all of the brain. Given that ischemic stroke in humans occurs mostly in the territory of the middle cerebral artery (MCA), experimental focal cerebral ischemia models, including permanent and transient types, have served as the most widely used tool in the stroke research field (Dorr et al., 2007; Mehta et al., 2007). In focal cerebral ischemia, there is no blood flow at the infarct core, but there is usually a gradient of blood flow from the inner core to the neighboring ischemic area due to the collateral circulation. In global cerebral ischemia, global blood flow is completely stopped or remarkably reduced, depleting the energy supply and hindering cerebral metabolism and function. The reperfusion of blood flow effectively treats acute stroke; however, it can also exacerbate tissue damage and limit the recovery of function. Oxidative stress plays a key role in the pathogenesis of cerebral ischemia-reperfusion injury.

(1) Permanent cerebral ischemia (pdMCAO and pMCAO): Technically, the MCA can be selectively occluded at a distal or proximal site, referred to as permanent distal MCA occlusion (pdMCAO) or permanent (proximal) MCA occlusion (pMCAO) cerebral ischemia model, respectively. The pdMCAO model produces highly reproducible ischemic cortical lesions that are predominantly restricted to the barrel regions of cortex, inducing definable sensorimotor deficits that closely mimics ischemic stroke in humans. Thus, it is believed to be one of the most predictable and useful stroke models, allowing researchers to look at long-term recovery with high survival rates (Doyle and Buckwalter, 2014). Permanent cerebral ischemia can also be generated by the intraluminal suture method used in MCAO (pMCAO).

(2) Transient focal cerebral ischemia (tMCAO): This model is easy to perform in a controlled manner. The intraluminal suture MCAO model in rats and mice is the most frequently used model. This model exhibits reproducible MCA region infarctions that depend on the shape, size, and insertion length of the thread, allowing reperfusion by retracting the suture. MCAO generates ischemic cell death in the striatum and overlying the frontal, parietal, temporal, and portions of the occipital cortex. MCAO also precipitates variable damage in the thalamus, cervicomedullary junction, substantia nigra, and hypothalamus. Ischemic brain injury widely affects diverse brain regions and leads to complex motor, sensory, autonomic, and cognitive deficits (Carmichael, 2005). Usually ischemia must be induced for 60–120 min to obtain reproducible infarct volumes in transient focal ischemia models. In contrast, focal ischemia for more than 3 h precludes reversibility (Kasner and Grotta, 1998; Sicard and Fisher, 2009).

(3) Global cerebral ischemia (GCI): global cerebral ischemia (GCI) during cardiac arrest results in selective and delayed neuronal death of pyramidal neurons in the hippocampal CA1 region, similar to the situation in humans, and consequent cognitive decline (Traystman, 2003; Ostrowski et al., 2016).

## Role Played by NRF2 During Cerebral Ischemia

In recent years, studies reported findings concerning the dynamic change of Nrf2 signaling, its functional importance, and its targeted intervention in cerebral ischemia. These findings provide insights into whether, when and how Nrf2 functions during brain injury. Accordingly, we mainly focused on the following questions. (1) What is the dynamic regulation of the Nrf2 signaling following cerebral ischemia? (2) Does the evidence from Nrf2^−/−^ mice support the functional importance of Nrf2 during ischemic injury? (3) Whether Nrf2 induction is protective against ischemic injury and is facilitative for recovery? Specific focus is given to the *in vivo* evidence in different rodent cerebral ischemia models. Then, the pitfalls and concerns of current Nrf2 experimental ischemic stroke studies are discussed, which would be valuable for future studies.

## The Dynamic Regulation of the NRF2 Signaling During Cerebral Ischemia

Normally, Nrf2 is largely localized in the cytoplasm and is maintained at a low basal level due to its binding affinity to Keap1. However, when cells are exposed to excessive oxidative stimuli during cerebral ischemia, Nrf2 is liberated from Keap1, translocates into the nucleus, and binds to the ARE sequence, thereby upregulating the expression of its target genes, which code cytoprotective proteins like anti-oxidative enzymes. In recent years, studies have provided substantial *in vivo* evidence of dynamic alternation of Nrf2 expression, as well as its target genes, and cellular and subcellular distribution of Nrf2 during different stages of cerebral ischemia. These findings, utilizing focal ischemia models with or without reperfusion in addition to global ischemia models, help us to identify the role of the Nrf2 regulatory network in the context of cerebral ischemia.

A number of studies with permanent cerebral ischemia models (pdMCAO and pMCAO) investigated the Nrf2/ARE pathway in response to ischemic insults (Table 1). Several permanent cerebral ischemia studies showed that, at 24 h after pMCAO, the protein expression levels of Nrf2 (total and nuclear) and its target antioxidant genes HO1 and SOD were upregulated in the ischemic cortex of mice or rats (Chang et al., 2013; Zhang J. et al., 2014; Zhao et al., 2014). The obvious increase of Nrf2 downstream antioxidant proteins can be sustained for at least 3 days following ischemia, revealed by the 1.8- to 3.6-fold increase in HO1, NQO1, SOD2, and GPx proteins after pdMCAO (Liu et al., 2018). The inflammatory factors IL-1β and IL-6 were dramatically increased in mouse brains for at least 48 h after pdMCAO (Clausen et al., 2017). These findings are supported by immunohistochemical analyses (Chen et al., 2012; Kao et al., 2013; Meng et al., 2014). Minimal Nrf2 and HO1 positive cells were detected in the sham group, indicating low baseline levels of Nrf2 and HO1 in the non-ischemic cortex areas, levels which increased significantly after pMCAO. Such signals were detected in neurons, astrocytes, and microglia, indicating concurrent expression of Nrf2 and HO1 in most cortical cells. On the contrary to above data, Nrf2 was reported to be significantly decreased at the mRNA level 6 h after pdMCAO, whereas the overall protein level was comparable to that under basal conditions (Clausen et al., 2017). A study reported a significantly decreased level of Nrf2 protein along with an increased level of antioxidant protein HO1 in rat cortex after pMCAO (Wang et al., 2018).

**Table 1:** Preclinical studies of Nrf2 in permanent cerebral ischemia models of mice and rats.

**Species**	**Genetic background; sex; age or weight**	**Treatment**	**Dosage/Administration route**	**Brain lesion/ Edema**	**Neurobehavioral deficits**	**Nrf2 Mechanism *(in vivo)***	**References**
**pdMCAO**							
**Findings supporting the role of Nrf2 pathway (with Nrf2**^**−/−**^ **mice)**							
Mouse	C57BL/6 (WT and **Nr*****f2***^−/−^); M; 10–18 wks	Korean Red Ginsen	• Pre;100 mg/kg; gavage; daily • For 7 d	• Infarct volume (3 d) ↓ • Above change is absent in Nrf2^−/−^ mice	• Open field • Cylinder (3, 7, 28 d) ↓ • Corner (3, 7, 14, 21, 28 d) ↓ • Above changes are absent in Nrf2^−/−^ mice	• HO1, NQO1, Gpx1 and SOD2 protein • Above changes are absent in Nrf2^−/−^ mice	Liu et al., 2018
Mouse	C57BL/6 (WT and ***Nrf2***^**−/−**^); M; 12 mo	-Epicatechin (EC)	• Pre; 15 mg/kg; gavage; 90 min before ischemia • Once	• Infarct volume (cortex; 7 d) ↓ • Above change is absent in Nrf2^−/−^ mice	• Adhesive removal (1 d) ↓ • Above change is absent in Nrf2^−/−^ mice • DigiGait	• NA	Leonardo et al., 2015
Mouse	C57BL/6 (WT and ***Nrf2***^**−/−**^); M; 2-3 mo	-Epicatechin (EC)	• Pre; 5, 10, or 15 mg/kg; gavage; 90 min before ischemia • Once	• Infarct volume (7 d; for 5 and 15 mg/kg) ↓ • Above change is absent in Nrf2^−/−^ mice	• Adhesive removal (1 d; for 5 and 10 mg/kg) ↓ • Above change is absent in Nrf2^−/−^ mice	• NA	Leonardo et al., 2013
Mouse	C57BL/6 (WT and ***Nrf2***^**−/−**^); 8-10wks	Carbon monoxide (CO)	• Post; 250 ppm at 1 L/min; inhalant; immediately after onset of ischemia • For 18 h	Infarct volume (cortex; 7 d) ↓	• Neurological deficits • Adhesive removal • Gross locomotor • Above change are decreased in Nrf2^−/−^ mice	• Nrf2 (nuclear ↑, cytoplasmic; at 75 KDa) and HO1 proteins	Wang B. et al., 2011
Mouse	C57BL/6 (WT and ***Nrf2***^**−/−**^); M; 6-8wks	Trichostatin A (TSA)	• Post; 1 mg/kg; i.p.; immediately and 6 h after onset of ischemia • Twice	• Infarct volume (cortex; 2 d) ↓ • Above change is absent in Nrf2^−/−^ mice	• Neurological deficits (2 d) ↓ • Above change is absent in Nrf2^−/−^ mice	• Nrf2 (nuclear ↑) and HO1, NQO1,GCLC proteins (*in vitro*) • Nrf2-ARE binding	Wang B. et al., 2011
Mouse/Rat	C57B/SV129 background ***Nrf2***^**−/−**^ mice, M, 10-16wks	• Tert-butylhydroquinone (tBHQ)	• Mouse: 1% tBHQ (w/w) food pellets	• Infarct volume (cortex; between WT and Nrf2^−/−^ mice; 1 d,7 d↓) • Above change is absent in Nrf2^−/−^ mice	• NA	• NQO1 ↓ in Nrf2^−/−^ mice	Shih et al., 2005
**Findings supporting the role of Nrf2 pathway (without Nrf2**^**−/−**^ **mice, indicated by Nrf2 protein nuclear translocation)**							
**NA**							
**Other findings involving the role of Nrf2 pathway**							
Mouse	C57BL/6; M; 8–10weeks	Monomethyl-fumarate (MMF)	• Post; 20 mg/kg; i.v.; 30 min after ischemia • Once	• Infarct size • Brain edema (1, 2 d) ↓	• Open field • Grip strength (1, 2 d) ↓ • Rotarod	• Nrf2 (total ↑) protein	Clausen et al., 2017
**pMCAO**							
**Findings supporting the role of Nrf2 pathway (with Nrf2**^**−/−**^ **mice)**							
**NA**							
**Findings supporting the role of Nrf2 pathway (without Nrf2**^**−/−**^ **mice, with Nrf2 protein nuclear translocation)**							
Mouse	CD-1 (ICR); M; 25–30 g	Paeonol (PN)	• Pre; 60 mg/kg; gavage; daily • For 3 d	• Infarct volume (24 h) ↓ • Brain edema (24 h) ↓	• Neurological deficits (24 h) ↓	• Nrf2 (nuclear ↑) and HO1 proteins • Nrf2 and HO1 mRNA	Zhao et al., 2014
Rat	SD; M; 250–300 g	Bicyclol	• Pre; 100 mg/kg; gavage; daily • For 3 d	• Infarct volume (24 h) ↓ • Brain edema (24 h) ↓	• Neurological deficits (24 h) ↓	• Nrf2 (nuclear ↑) and HO1, SOD proteins	Zhang J. et al., 2014
Rat	SD; M; 230–270 g	Recombinant human erythropoietin (rhEPO)	• Post; 5,000 IU/kg; i.p.; 2 h after onset of ischemia • Once	• Infarct volume (24 h) ↓ • Brain edema (24 h) ↓	• NA	• Nrf2 (nuclear ↑; at 70KDa) and HO1 proteins	Meng et al., 2014
Rat	SD; M; 250–280 g	Nobiletin	• Pre and Post; 25 mg/kg; i.p.; 3 d before AND once immediately after onset of ischemia; daily • For 4 d	• Infarct volume (cortex and striatum; 24 h) ↓ • Brain edema (24 h) ↓	• Neurological deficits (24 h) ↓	• Nrf2 (nuclear ↑) and HO1 proteins • Nrf2 and HO1 (IHC)	Zhang L. et al., 2016
**Other findings involving the role of Nrf2 pathway**							
Rat	SD; M; 300-350 g	Docosahexaenoic acid (DHA)	• Pre; 500 nmol/kg; i.p.; daily • For 3 d	• Infarct volume (3 d) ↓ • Brain edema (3 d) ↓	• Neurological deficits(3 d) ↓	• Nrf2 (total ↑) and HO1 proteins	Chang et al., 2013
Rat	SD; M/F; 250–280 g	HP-1c	• Post; 1 mg/kg; i.v.; 4 h after MCAO and the next 2 d; daily • 3 times	• Infarct volume (2 d) ↓	• Neurological deficits (3 d) ↓ • Rotarod (3, 7, 14, 21 d) • Corner (3, 7 d) ↓ • Open field (1 d) ↓	• Nrf2 (total ↑; at 100 kDa) and HO1 proteins (*in vitro*)	Wang et al., 2018
Rat	SD; M; 230–280 g	Octreotide (OCT)	• Post; 100 mg/kg; i.p.; immediately after onset of ischemia • Once	• Infarct volume (24 h) ↓ • Brain edema (24 h) ↓	• Neurological deficits (24 h) ↓	• Nrf2 (total ↑) and HO1 proteins • Nrf2 ↑ and HO1 (IHC) • SOD activity	Chen et al., 2012
Rat	SD; M; 250–300 g	Tetramethylpyrazine (TMP)	• Pre and Post; 20 mg/kg; i.p.; 30 min before and 60 min after onset of ischemia • Twice	• NA	• NA	• Nrf2 (total ↑) and HO1 proteins	Chang et al., 2015
Rat	SD; M; 300-350 g	Tetramethylpyrazine (TMP)	• Pre and Post; 20 mg/kg; i.p.; 30 min before and 60 min after onset of ischemia • Twice	• Infarct volume (3 d) ↓ • Brain edema (3 d) ↓	• Neurological deficits (3 d) ↓	• Nrf2 (total ↑) and HO1 proteins • Nrf2 and HO1 (IHC)	Kao et al., 2013
Rat	SD; M; 8 wks; 220-260 g	Isoquercetin	• Post; 50 mg/kg; i.v.; daily • For 7 d	• Infarct volume (24 h) ↓	Neurological deficits (24 h) ↓	• Nrf2 mRNA/protein (total ↑)	Chen M. et al., 2017
Mouse	C57BL/6J; M; 3-4 mo; 25–30 g	Tert-butylhydroquinone (tBHQ)	• Pre; 0.582, 3.34, or 33.4 mg/kg; i.p.; started 24 h before ischemia; once every 12 h • 3 times	• Infarct volume (cortex; striatum; hemisphere)	• Neurological deficits (1 d) ↑ • Mortality ↑	• NA	Sun et al., 2016

*MCAO, middle cerebral artery; pdMCAO, permanent distal middle cerebral artery occlusion; pMCAO, permanent (proximal end of) middle cerebral artery occlusion that is generated by the intraluminal suture MCAO; GCI, global cerebral ischemia; i.p., intraperitoneal; i.v., intravenous; i.c.v., intracerebroventricular; Pre, pretreatment; Post, posttreatment; KDa, kilodalton that indicates Nrf2 protein molecular weight by Western blot; the changes in brain lesion, edema, neurological deficits, and mRNA/protein expression level (↑ or ↓, increase or decrease at indicated time point; no label at indicated time point, no significant difference); h, hour; d, day; wk, week*.

It appears that the majority of tMCAO studies support the activation of the Nrf2/ARE pathway in response to focal cerebral ischemia and perfusion (Tables 2, 3). Because the ischemia-reperfusion injury measured by infarct volume is widely accepted to be most severe at 24 h following tMCAO, most studies investigated the Nrf2 pathway by Western blot (WB) or immunohistological analyses at that time point, with some extending to earlier (2–8 h) or later stages (3–14 d). In these studies, the Nrf2 pathway was examined in different ischemic brain tissues including the cortex, hemisphere, hippocampus, striatum, and cerebellum. The researchers selected 1–2 h ischemia when designing these tMCAO ischemia-reperfusion animal models. We summarized these studies together in order to provide an overview of this field. Most studies measured total Nrf2 level in the ischemic cortex or brain at 24 h after tMCAO (1–2 h); studies subjects showed Nrf2 upregulation at the mRNA level (Li et al., 2013; Guo et al., 2014) and protein level (up to 2–3 fold higher) (Han et al., 2014; Li et al., 2015; Peng et al., 2015; Shi et al., 2015; Cai et al., 2017; Miao et al., 2018; Shang et al., 2018). Such an increase in Nrf2 protein might begin at 8 h (Cai et al., 2017) and be sustained over 3–14 days (Ding et al., 2015; Lin et al., 2016; Bai et al., 2017; Shang et al., 2018). Following nuclear translocation, Nrf2 protein accumulated in the nucleus (Li et al., 2013; Lv et al., 2017) and increased the Nrf2-binding activity to the ARE. Studies presented that the high nuclear Nrf2 protein level (Ding et al., 2015; An et al., 2018) and increased DNA binding activity of Nrf2 with the ARE can be observed, at 7 days after tMCAO (Li et al., 2014). The Nrf2 target antioxidant protein HO1 (up to 3 fold) (Li et al., 2013; Zhang M. et al., 2014; Hua et al., 2015; Peng et al., 2015; Lv et al., 2017; Yang et al., 2017), NQO1(Miao et al., 2018), GCLC and LCLM (Han et al., 2014; Shi et al., 2015) were also upregulated. Several reports showed that the higher expression level of HO1 can continue over 2–14 days (Zhang M. et al., 2014; Ding et al., 2015; Lin et al., 2016). At 24 h after tMCAO, the total and nuclear Nrf2 proteins levels in the ischemic hippocampus also implied significantly higher expression levels (Shi et al., 2015; Lou et al., 2016), which may continue for at least 2 days. In addition, it was reported by immunohistochemistry that, Nrf2 levels in the peri-infarct regions began to show a significant increase at 2 h with a peak at 8 h of reperfusion after 1 h tMCAO, and its target antioxidative proteins such as thioredoxin, glutathione, and HO1 showed significant increases at 24–72 h (Tanaka et al., 2011). Another study presented that Nrf2 immunoreactivity was detectable in the neurons, endothelial cells, astrocytes, and microglia in the peri-infarct area at 7 days after 1 h tMCAO (Shang et al., 2018). A luciferase mouse model, a Keap1-dependent oxidative stress detector, was employed to visualize the time-dependent Nrf2 expression from brain ischemia onset through 7 days after tMCAO (Takagi et al., 2014; Nakano et al., 2017). The *in vivo* optical signals of Nrf2 expression were not detected in the earliest stages but peaked at 24 h after ischemia. Such Nrf2 expression was mainly detected in in the penumbra area, largely localizing inside neurons and astrocytes (Srivastava et al., 2013; Takagi et al., 2014). By quantifying Nrf2 labeled with green fluorescent protein (GFP), researchers have found that Nrf2 and HO1 exhibited higher protein levels in the ischemic cortex at 4–48 h and striatum at 24 h after ischemia, which are consistent with the findings above. In contrast, some studies present the results that indicate suppression of the Nrf2/ARE pathway during tMCAO. At 24 h after tMCAO, the total (Wang L. et al., 2012; Pang et al., 2016; Wang et al., 2016, 2018; Chumboatong et al., 2017; Janyou et al., 2017) and nuclear (Wicha et al., 2017) Nrf2 proteins were found to be downregulated, and the same was observed with HO1 (Wang L. et al., 2012; Pang et al., 2016; Wang et al., 2016, 2018; Chumboatong et al., 2017; Wicha et al., 2017) and NQO1 (Janyou et al., 2017). On the contrary to above findings, several reports found that no change was detected in the markers above (Wu L. et al., 2013; Zhao et al., 2016;Wu G. et al., 2017; Zhang W. et al., 2018).

**Table 2:** Preclinical studies of Nrf2 in transient cerebral ischemia models of mice and rats (MCAO, 40 min-1.5 h).

**Model**	**Species**	**Genetic Background; Sex; Age or Weight**	**Treatment**	**Dosage/Administration route**	**Brain lesion/Edema**	**Neurobehavioral deficits**	**Nrf2 Mechanism (*in vivo*)**	**References**
**MCAO (40 min-1.5 h)**								
**Findings supporting the role of Nrf2 pathway (with Nrf2**^**−/−**^ **mice)**								
*MCAO (40 min)*	Mouse	C57BL/6, ***Nrf2***^**−/−**^, and Cx3cr1GFP/+; M; 23–30 g	3H-1,2-Dithiole-3-thione (D3T)	• Post; 50 mg/kg; i.p.; at 3 h after MCAO • Once	• Infarct volume (48 h) • Brain edema (48 h) • Above changes are decreased in Nrf2^−/−^ mice	• Neurological deficits (48 h) ↓ • Above changes are absent in Nrf2^−/−^ mice	• Nrf2 (total ↑) and HO1 proteins (*in vitro*) • Above changes are absent in Nrf2^−/−^ mice • Nrf2 (IHC)	Kuo et al., 2017
*MCAO (1 h)*	Mouse	C57BL/6J WT, ***Nrf2***^**−/−**^; M; 7–11wks;	Resveratrol	• Pre: 10 mg/kg; i.p.; 48 h before MCAO • Once	• Infarct volume (24 h) ↓ • Above change is absent in Nrf2^−/−^ mice	• NA	• NQO1, SOD2 proteins • Above changes are decreased in Nrf2^−/−^ mice	Narayanan et al., 2015
*MCAO (1 h)*	Mouse	C57BL/6J: ***Nrf2***^**−/−**^ and WT; M; 8–10wks	Lentiviral transfection (for SIRT6 overexpression)	• Pre; 2.5 μl (10^9^ infectious units/ml); i.c.v.; 2 wks before MCAO • Once	• Infarct volume (24 h) ↓ • Above change is absent in Nrf2^−/−^ mice	• Neurological deficits (24 h) ↓ • Above change is absent in Nrf2^−/−^ mice	• Nrf2 (total ↑) and HO1 proteins	Zhang et al., 2017
*MCAO (1 h)*	Mouse	C57BL/6: WT and ***Nrf2***^**−/−**^; 20–25 g, 8 to 10 wks	Dimethyl fumarate (DMF), Monomethyl fumarate (MMF)	• Post: 30, 45 mg/kg (better); i.p.; 15 min before reperfusion twice a day • For 7 d	• Infarct volume (3,7 d) ↓ • Brain edema (3 d) ↓ • Above changes are absent in Nrf2^−/−^ mice	• Neurological deficits (3,7 d) ↓ • Above change is absent in Nrf2^−/−^ mice	• Nrf2 (total ↑) and HO1 proteins	Yao et al., 2016
*MCAO (1 h)*	Mouse	WT and ***Nrf2***^**−/−**^; M; 8–10 wks	Tanshinone IIA (TSA)	• Post; 25 mg/kg; i.p.; 10 min after MCAO • Once	• Infarct volume (72 h) • Above change is reduced in Nrf2^−/−^ mice	• Neurological deficits (72 h) ↓ • Above change is absent in Nrf2^−/−^ mice	• Nrf2 (nuclear ↑) protein • Nrf2 mRNA	Cai et al., 2017
*MCAO (1 h)*	Mouse	ICR background ***Nrf2***^**−/−**^ and WT; 25–28 g	Ursolic acid (UA)	• 130 mg/kg; i.p.; immediately after MCAO; once	• Infarct volume (24 h) ↓ • Above change is absent in Nrf2^−/−^ mice	• Neurological deficits (24 h) • Above change is reduced in Nrf2^−/−^ mice	• Nrf2 (nuclear ↑, cytoplasmic ↓) and HO1 proteins • Nrf2 and HO1 mRNA	Li et al., 2013
*MCAO (1.5 h)*	Mouse	HO1^−/−^, ***Nrf2***^**−/−**^ and WT; M; 7–8 wks; 20–25 g	Epicatechin (EC)	• Pre: 2.5 (no effect), 5, 15, 30 mg/kg (best); oral; 90 min before; once • Post: 30 mg/kg EC was administered at 3.5 h (better) or 6 h (no effect) after MCAO	• Infarct volume (pre; 24 h) ↓ • Above change is absent in Nrf2^−/−^ mice • Infarct volume (post; 72 h) ↓	• Neurological deficits (pre; 24 h) • Above change is reduced in Nrf2^−/−^ mice • Neurological deficits (post; 72 h)	• Nrf2 (nuclear ↑, cytoplasmic) and HO1 proteins	Shah et al., 2010
*MCAO (1.5 h)*	Mouse	CD1 background ***Nrf2***^**−/−**^ and WT; F; 20–25 g	Tert-butylhydroquinone (t-BHQ)	• NA	• Infarct volume (between Nrf2^−/−^ and WT, 24 h) • Above change is reduced in Nrf2^−/−^ mice	• Neurological deficits (between Nrf2^−/−^ and WT; 24 h) • Above change is reduced in Nrf2^−/−^ mice	• NA	Shah et al., 2007
**Findings supporting the role of Nrf2 pathway (without Nrf2**^**−/−**^ **mice, indicated by Nrf2 protein nuclear translocation)**								
*MCAO (1 h)*	Mouse	ICR; M; 24–27 g	Isorhamnetin (Iso)	• Post; 5 mg/kg; i.p.; immediately at the onset of reperfusion; daily; • Twice	• Infarct volume (48 h) ↓ • Brain edema (48 h)	• Neurological deficits (48 h) ↓ • Rotarod (48 h)	• Nrf2 (nuclear ↑, cytoplasmic) and HO1 proteins	Zhao et al., 2016
*MCAO (1.5 h)*	Mouse	C57BL/6; M; 25–30 g; 10–12 wks	Epigallocatechin-3-gallate (EGCG)	• Post; 50 mg/kg; i.p.; immediately after; daily • For 7 d	• Infarct volume (7 d) ↓	• Neurological deficits (3, 7 and 14 d) ↓	• Nrf2 (nuclear ↑) protein • Nrf2 (IHC)	Bai et al., 2017
*MCAO (1.5 h)*	Rat	SD; M; 280–300 g	Diterpene ginkgolides meglumine injection (DGMI)	• Post; 1, 3 and 10 mg/kg, iv, at the onset of reperfusion and 12 h after reperfusion	• Infarct volume (3 and 10 mg/kg, 24 h) ↓	• Neurological deficits (1, 3 and 10 mg/kg dose-dependent, 24 h) ↓	• Nrf2 (nuclear ↑) and HO1	Zhang W. et al., 2018
*MCAO (1 h)*	Rat	SD; M; 3 mo	5-methoxyindole-2-carboxylic acid (MICA)	• Pre; diet supplemented with 0.33% MICA (200 mg/kg/d) for 4 wks before MCAO; i.p. injection (200 mg/kg body weight) once per day • For seven days until 24 h before MCAO	Infarct volume (24 h) ↓	• NA	• Nrf2 (nuclear ↑) and NQO1 proteins	Wu et al., 2017b
**Other findings involving the role of Nrf2 pathway**								
*MCAO (1 h)*	Mouse	C57BL/6J background WT and SHPS-1 mutant (MT); M; 10–12 wks	Src homology 2 domain–containing protein tyrosine phosphatase substrate−1 (SHPS-1)	• NA	• Infarct volume (24 h) ↓	• Neurological deficits (72 h) ↓	• Nrf2 (total ↑; at 98 KDa) and HO1 proteins	Wang B. et al., 2011
*MCAO (1 h)*	Mouse	C57BL/6 J; M; 22–25 g	MiR-93 antagomir	• Pre; 7 μl (at 100 μm); i.c.v.; 10 min before MCAO • Once	• Infarct volume (24 h) ↓	• Neurological deficits (24 h) ↓	• Nrf2 (total ↑) and HO1 proteins	Wang et al., 2016
*MCAO (1 h)*	Mouse	ICR; M; 6 wks old, 23–25 g	Tocovid	• 200 mg/kg/d orally once a day for 1 mo before MCAO	• Infarct volume (1, 3 d) ↓	• Bederson score (pre,1,3,7 d), • Rotarod (pre,1,3,7 d) • Corner (pre,1,3,7 d)	• Nrf2 (total ↑) protein • Nrf2 (IHC)	Shang et al., 2018
*MCAO (1 h)*	Mouse	C57BL/6 J	Gastrodin (GAS)	• Post: 10, 50, 100 mg/kg; i.p.; onset of cerebral reperfusion; • Once daily for 7 d	• Infarct volume (medium or high-dose, 24 h and 7 d) ↓	• Neurobehavioral scores (1, 7 d) ↓	• Nrf2 (total ↑; at 68 KDa) and HO1 proteins	Peng et al., 2015
*MCAO (1 h)*	Mouse	C57BL/6; F; 12–15 wks	Estradiol (EST)	• 0.05 mg; pellets; subcutaneous implantation; before; for 21 d	• Brain edema (24 h) ↓	• Neurological deficits (mNSS, 24 h) ↓	• Nrf2 (total ↑) and NQO1 proteins	Li et al., 2017
*MCAO (1 h)*	Rat	SD; M; 60–80 d old, 260–300 g	Tert-butylhydroquinone (tBHQ)	• Pre; 16.7 mg/kg; i.p. injection at intervals of 8 h before MCAO • Three times	• Infarct volume (24 h)	• Neurological deficits (24 h) ↓	• Nrf2 (total ↑) protein	Hou et al., 2018
*MCAO (70 min)*	Rat	SD; M; 250–330 g	Sulforaphane	• 5 mg/kg; i.p.; 1 h before; once	• Infarct volume (24 h, 72 h)	• Neurological deficits (24 h) ↓	• Nrf2 (total content ↑) and NQO1 proteins • HO1 (IHC)	Alfieri et al., 2013
*MCAO (1 h)*	Rat	SD; M; 3 mo	5-methoxyindole-2-carboxylic acid (MICA)	• Post; 100 mg/kg;i.p.; at the onset of reperfusion • Once	• Infarct volume (24 h) ↓	• NA	• Nrf2 (total ↑) and NQO1 proteins	Wu et al., 2018
*MCAO (1.5 h)*	Rat	SD; F; 250–300 g	Genistein	• Pre; 10 mg/kg, i.p., once daily • For 2 wks	• Infarct volume (72 h) ↓	• Neurological deficits (72 h) ↓	• Nrf2 (total ↑) and NQO1 proteins	Miao et al., 2018
*MCAO (1 h)*	Rat	SD; M; 60–80 d old, 240–300 g	Glycogen synthase kinase 3β (GSK-3β)	• Pre: 7 μl (2 μg/μl); i.c.v.; 48 h before MCAO • Once	• NA	• NA	• Nrf2 (total) and NQO1, HO1 proteins • NQO1, HO1 mRNA	Chen X. et al., 2016
*MCAO (1 h)*	Rat	SD; M; 250–280 g	siRNA targeting sulfiredoxin1 (Srxn1)	• Pre; i.c.v.; 24 h before MCAO • Once	Infarct volume (24 h)	• Neurological deficits (24 h) ↑	• Nrf2 (total ↓) and NQO1 proteins	Wu et al., 2017a
*MCAO (1 h)*	Rat	SD; M; 270–310 g	Thioredoxin-1 siRNA	• Pre: 10 μl (2 μg/μl); i.c.v.; 24 h before MCAO • Once	• Infarct volume (24 h) • Brain edema (24 h)	• Neurological deficits (24 h) ↓	• Nrf2 (total ↓) protein	Li et al., 2015
*MCAO (1 h)*	Rat	SD; M; 280–310 g	Sevoflurane	• Post: 2.6% for 1 h; inhalation; immediately at onset of reperfusion • Once	• Infarct volume (72 h)	• Neurological deficits (12, 24, 48, and 72 h) n	• Nrf2 (total ↓) and NQO1 • Nrf2-DNA binding activities	Li et al., 2014
*MCAO (70 min)*	Rat	SD; M; 250–300 g	Nrf2 inducer D, L-sulforaphane	• 5 mg/kg; i.p.; 1 h before MCAO • Once	• NA	• NA	• Nrf2 (IHC)	Srivastava et al., 2013
*MCAO 1 h*	Rat	SD; M; aged 8–9 wks; 300–350 g	• MicroRNA (miR-142-5p)	• NA	• NA	• NA	• Nrf2 mRNA	Wang et al., 2017
*MCAO (1 h)*	Rat	Wistar; M; 250–280 g	Danhong	• 0.9, 1.8 ml/kg; i.p.; 30 min before ischemia, with reperfusion and 24, 48, 72 h after ischemia	• Infarct volume (72 h) ↓ • Brain edema (72 h) ↓	• Neurological deficits (72 h)	• Nrf2 and HO1, NQO1 mRNA	Guo et al., 2014
*MCAO (1.5 h)*	Rat	SD; M; 180–220 g	Lactulose	• Pre; 0.25 g/kg; gavage; at start of ischemia • Once	• Infarct volume (24 h) ↓	• Neurological deficits (24 h) ↓ • Morris water maze ↓	• Nrf2 mRNA and activity • SOD activity	Zhai et al., 2013
*MCAO (1.5 h)*	Rat	SD; M; 260–290 g	β-caryophyllene (BCP)	• Pre; 34, 102, 306 mg/kg (best); gavage; once a day • For 7 d	• Infarct volume (24 h) ↓	Neurological deficits (24 h) ↓	• Nrf2 (total ↑) and HO1 mRNA	Lou et al., 2016
*MCAO (1.5 h)*	Rat	SD; M; 260–280 g	Neural stem cells (NSCs)	• Post; four 1.0 μl deposits of single-cell suspension in Dulbecco's PBS (10^5^ cells per ul); along the anterior-posterior axis into the cortex; 6 h after stroke	• Infarct volume (cortex, 28 d) ↓	• Rotarod (1–28 d) • Beam-balance (28 d) ↓	Nrf2 mRNA	Sakata et al., 2012
*MCAO (1.5 h)*	Rat	Wistar; M; 250–280 g	Xueshuantong injection (Lyophilized, XST)	• Post; 25, 50,100 mg/kg; i.p.; 1 h after reperfusion, once a day • For 3 or 7 d	• NA	• Modified neurological severity (Mnss, 1, 3, and 7 d) ↓	• Nrf2 and HO1, NQO1 mRNA	Guo et al., 2018
*MCAO (1 h)*	Rat	SD; F; 300–350 g	p-hydro-xybenzyl alcohol (HBA)	• Pre; 25 mg/kg BW; i.m. with sesame oil; 3 d before • Once	• Infarct volume (cortex and striatum, 24 h) ↓	• Modified neurological severity score (mNSS) at 1, 7, 14, 21, and 28 d • Functional deficits from 7 d ↓	• Nrf2 DNA (PCR)	Kam et al., 2011
*MCAO (1 h)*	Rat	SD; M; 230–270 g	Curcumin	• Post; 300 mg/kg; i.p.; 1 h after MCAO • Once	• Infarct volume (24 h) ↓	• NA	• Nrf2-DNA binding activity	Wu J. et al., 2013
*MCAO (45 min)*	Mouse	OKD48 transgenic mice; M/F; 23–28 g	NA	• NA	• Infarct volume (12 h, 1, 3, 7 d)	• NA	• Nrf2 (IF)	Nakano et al., 2017
*MCAO (1 h)*	Mouse	ICR; M; 34–38 g; 8 wks	NA	• NA	• NA	• NA	• Nrf2 (IHC)	Tanaka et al., 2011
*MCAO (1 h)*	Rat	Hannover-Wistar; M; 250–350 g	Recombinant human erythropoietin (rhEpo)	• Post; 5000 IU/kg; i.p. immediately or 3 h after MCAO • Once	• Infarct volume (3, 24 h)	• NA	• Nrf2 (IHC)	Mrsic-Pelcic et al., 2017
*MCAO (1 h)*	Rat	Wistar; M; 10 wks; 250–300 g	Curcumin	• Post; 300 mg/kg; i.p.;Post; 300 mg/kg; i.p.; 30 min after MCAO • Once	• Infarct volume (d1) ↓ • Brain edema (d1) ↓	• Neurological deficits (d1) ↓	• Nrf2 (IHC)	Li et al., 2016
*MCAO (1 h)*	Mouse	Transgenic fatty acid metabolism-1 (*fat-1*) gene mice; C57BL/6; M	Omega-3 fatty acids (n-3 PUFAs) by fish oil (FO) diet	• Pre: 5% (w/w) was added to the regular diet, which increased the n-3 PUFA from 0.34 to 1.5%, and decreased the n-6:n-3 PUFA ratio from 5:1 to 1:1; oral; before; daily; for 6 wks	• Infarct volume (48 h) ↓	• Neurological deficits (48 h) ↓ • Dietary supplementation in Corner, Rotarod (7 d) d supplementation in Corner, Rotarod	HO1 protein	Zhang M. et al., 2014
*MCAO (1 h)*	Mouse	C57BL/6; M; 8-−0 wks	Dimethyl fumarate (DMF)	• Pre: 15 mg/kg; i.p.; twice a day for 3 d before stroke	• Infarct volume (4 h, 24 h) • Brain edema (4 h, 24 h) ↓	• NA	• HO1, NQO1,GCLC and GCLM mRNA	Kunze et al., 2015
*MCAO (1.5 h)*	Rat/ Mouse	Wistar (Osmotic pump studies) and SD, 250–350 g;	Tert-butylhydroquinone (tBHQ)	• Rat: 1 mM, i.c.v., osmotic mini-pump delivery (1 μl/h for 4 d), MCAO after 3 • d; i.p. in later experiments, 3.33 or 16.7 mg/kg, before; three times by 8 h intervals	• Rat: Infarct volume (cortex, 24 h) ↓	• Rat: tBHQ Neurological deficits (24 h to 1 mo) • Sensorimotor deficits (since 4 d) ↓	NA	Lou et al., 2016

*MCAO, middle cerebral artery; pdMCAO, permanent distal middle cerebral artery occlusion; pMCAO, permanent (proximal end of) middle cerebral artery occlusion that is generated by the intraluminal suture MCAO; GCI, global cerebral ischemia; i.p., intraperitoneal; i.v., intravenous; i.c.v., intracerebroventricular; Pre, pretreatment; Post, posttreatment; the changes in brain lesion/edema and neurobehavioral deficits/Nrf2 protein expression level rf2 protein expression leveledema and neurobehavioral deficitseatmest, posttreatmeatme; IF, Immunofluorescence; KDa, kilodalton that indicates Nrf2 protein molecular weight by Western blot; the changes in brain lesion, edema, neurological deficits, and mRNA/protein expression level (↑ or ↓, increase or decrease at indicated time point; no label at indicated time point, no significant difference); h, hour; d, day; wk, week*.

**Table 3:** Preclinical studies of Nrf2 in transient cerebral ischemia models of mice and rats (MCAO, 2 h).

**Species**	**Genetic Background; Sex; Age or Weight**	**Treatment**	**Dosage/Administration route**	**Brain lesion/Edema**	**Neurobehavioral deficits**	**Nrf2 Mechanism (*in vivo*)**	**References**
**MCAO (2 h)**							
**Findings supporting the role of Nrf2 pathway (with Nrf2**^**−/−**^ **mice)**							
Mouse	WT and **Nr*****f2**^**−/−**^*; 12 wks	S-allyl cysteine (SAC)	• Pre; 300 mg/kg; i.p.; 30 min before MCAO • Once	• Infarct volume (1 d) ↓ • Above change is absent in Nrf2^−/−^ mice	• Neurological deficits (1 d) ↓ • Above change is absent in Nrf2^−/−^ mice	• Nrf2 (nuclear ↑) and HO1, GCLC,LCLM proteins	Shi et al., 2015
Mouse	WT and **Nr*****f2**^**−/−**^*; M; 30-35 g	Hydrogen sulfide (H2S)	• Pre; 40 ppm; inhalant; 7 d before MCAO; daily	• Infarct volume (1 d) ↓	• Neurological deficits (1 d) ↓ • Above change is absent in Nrf2^−/−^ mice • Morris water maze ↓	• Nrf2 (nuclear ↑) protein	Ji et al., 2016
**Findings supporting the role of Nrf2 pathway (without Nrf2**^**−/−**^ **mice, indicated by Nrf2 protein nuclear translocation)**							
Rat	SD; M; 220-240 g	Protocatechualdehyde (PCA)	• Pre; 40 mg/kg; i.v.; 1 h before reperfusion • Once	• Infarct volume (1 d) ↓	• Neurological deficits (1 d) ↓	• Nrf2 (nuclear ↑) and cytoplasmic HO1	Guo et al., 2017
Rat	SD; M; 200-250 g	Procyanidin B2 (PB)	• Post; 40 mg/kg; gavage; 3 h after MCAO then daily • For 14 d	• Brain edema (2 d) Infarct volume (2 d) ↓	• Neurological deficits (7, 11, 14 d) ↓ • Rotarod (7, 11, 14 d)	• Nrf2 (nuclear ↑) and HO1, NQO1, GSTa proteins	Wu et al., 2015
Rat	Wistar; M; 250-300 g	Hexahydrocurcumin (HHC)	• Post; 40 mg/kg; i.p.; immediately after MCAO • Once	• Infarct volume (1 d) ↓	• Neurological deficits (1 d) ↓	• Nrf2 (nuclear ↑; at 100 KDa) and HO1, NQO1 proteins • SOD activity	Wicha et al., 2017
Rat	SD; M; 220-250 g	Alpha-lipoic acid (α-LA)	• Post; 40 mg/kg; i.v.; immediately after reperfusion • Once	• Infarct volume (1 d) ↓ • Brain edema (1 d) ↓	• Neurological deficits (1 d) ↓	• Nrf2 (nuclear ↑, cytoplasmic ↓; at 68 KDa) and HO1 proteins	Lv et al., 2017
Rat	Wistar; M; 6 mo; 270–290 g	Hispidulin	• Post; 50 mg/kg; i.p.; onset of MCAO then daily • For 7 d	• Infarct volume (7 d) ↓ • Brain edema (7 d) ↓	• Neurological deficits (2, 3, 5, 7 d) ↓ • Beam-walking (2, 3, 5 7 d) ↓ • Morris water maze (1, 2, 3, 5, 7 d) ↓	• Nrf2 (nuclear ↑, cytoplasmic ↑) protein • Nrf2 mRNA	An et al., 2018
Rat	SD; 280–320 g	Corilagin	• Post; 30 mg/kg; i.p.; 3 h after MCAO then daily • For 7 d	• Infarct volume (7 d) ↓	• Neurological deficits (7 d) ↓	• Nrf2 (nuclear ↑) protein, Nrf2 phosphorylation	Ding et al., 2017
Rat	SD; M; 240-280 g	Gualou Guizhi granule (GLGZG)	• Post; 3 g/kg; gavage; daily • For 7 d	• NA	• NA	• Nrf2 (nuclear ↑) and HO1, NQO1 proteins	Zhang Y. et al., 2018
Rat	SD; M; 3 mo; 210-230 g	Isoquercetin (Iso)	• Post; 20 mg/kg; gavage; after MCAO; daily for 3 d	• Infarct volume (3 d) ↓ • Brain edema (3 d) ↓	• Neurological deficits (3 d) ↓	• Nrf2 (nuclear ↑, cytoplasmic ↓) protein	Dai et al., 2018
Rat	SD; M; 10 mo; 350-400 g	Myricetin	• Pre and Post; 20 mg/kg; gavage; 2 h before MCAO then daily • For 2 d	• Infarct volume (1 d) ↓	• Neurological deficits (5, 7, 9, 11, 14 d) ↓ • Foot-fault (7, 9, 11, 14 d) ↓ • Modified balance beam (5, 7, 9, 11, 14 d) ↓ • Adhesive-removal somatosensory (7, 9, 11, 14 d) ↓ • Morris water maze (10 d) ↓ • Probe test (14 d) ↓	• Nrf2 (nuclear ↑, cytoplasmic) and HO1 proteins	Wu et al., 2016
Rat	SD; M; 250-280 g	Lipoxin A4 (LXA4)	• Post; 1 nmol; i.c.v.; immediately after MCAO • Once	• Infarct volume (1 d) ↓	• Neurological deficits (1 d) ↓	• Nrf2 (nuclear ↑, total ↑) protein	Wu L. et al., 2013
Rat	SD; M; 230-270 g	Huang-Lian-Jie-Du-Decoction (HLJDD)	• Pre; 20 mg/kg; gavage; daily • For 7 d	• Infarct volume (1 d) ↓	• Neurological deficits (1 d) ↓ • Mortality ↓	• Nrf2 (nuclear ↑, cytoplasmic ↓) and HO1 proteins	Zhang Q. et al., 2016
Rat	Wistar; M; 220-250 g	Mangiferin	• Pre and Post; 100 mg/kg; gavage; 3 times before and once at 2 h after MCAO onset; daily • For 4 d	• Infarct volume (1 d) ↓ • Brain edema (1 d) ↓	• Neurological deficits (1 d) ↓	• Nrf2 (nuclear ↑, cytoplasmic ↓) protein	Yang et al., 2016
**Other findings involving the role of Nrf2 pathway**							
Mouse	ddY WT and C57BL/6 OKD-V, OKD-LUC; M; 8–12 wks	Bardoxolone methyl (BARD)	• Pre; 0.6 or 2 mg/kg; i.v.; immediately before reperfusion • Once	• Infarct volume (1 d) ↓	• Neurological deficits (1 d) ↓ • Grid walk (1 d) ↓	• Nrf2 (total ↑) and HO1 • Nrf2 (IF)	Takagi et al., 2014
Mice	NA	Artesunate	10–40 mg/kg	• infarct volume (22 h) ↓	• NA	• Nrf2 (total ↑) protein	Lu et al., 2018
Mouse	ddY; M; 5–8 wks; 22–28 g	RS9	• Post; 0.2 mg/kg; i.p.; immediately after reperfusion • Once	• Infarct volume (1 d)	• Neurological deficits (1, 3, 5, 7 d) • Grid walk (1, 3, 5, 7 d) 1, • Mortality ↓	• Nrf2 (total ↑) protein	Yamauchi et al., 2016
Rat	SD; M/F; 250–280 g	HP-1c	• Post; 1 mg/kg; i.v.; 4 h after MCAO then daily • For 2 d	• Infarct volume (2 d) ↓	• Neurological deficits (3 d) ↓ • Rotarod (3, 7, 14, 21 d) c • Corner (3, 7 d) ↓ • Open-field ↓	• Nrf2 (total ↑; at 100 KDa) and HO1 proteins	Wang et al., 2018
Rat	SD; M; 230–280 g	Resveratrol	• Pre; 15 or 30 mg/kg; i.p.; daily • For 7 d	• Infarct volume (1 d) ↓ • Brain edema (1 d) ↓	• Neurological deficits (1 d) ↓	• Nrf2 (total ↑) protein	Ren et al., 2011
Rat	SD; M; 220–280 g	Phloretin	• Pre; 80 mg/kg; i.p.; daily • For 14 d	• Infarct volume(1 d) ↓ • Brain edema (1 d) ↓	• Neurological deficits (1 d) ↓	• Nrf2 (total ↑, at 61 KDa) protein and mRNA	Liu Y. et al., 2015
Rat	Wistar; M; 280–300 g	Dihydrocapsaicin (DHC)	• Pre; 5 or 10 mg/kg; i.p.; 15 min before reperfusion • Once	• Infarct volume (1 d) ↓	• Neurological deficits (1 d) ↓	• Nrf2 (total ↑; at 68 KDa) and NQO1 proteins • SOD and GPx activity	Janyou et al., 2017
Rat	Wistar; M; 220–250 g	Agomelatine	• Pre; 40 mg/kg; i.p.; 1 h before MCAO • Once	• Infarct volume (1 d) ↓	• Neurological deficits (1 d) ↓	• Nrf2 (total ↑; at 57 KDa) and NQO1 proteins • SOD and GPx activity	Chumboatong et al., 2017
Rat	SD; M; 270–320 g	(–)-Epigallocatechin gallate (EGCG)	• Pre; 40 mg/kg; i.p.; daily • For 3 d	• Infarct volume (1 d) ↓	• Neurological deficits (1 d) ↓	• Nrf2 (total ↑) and HO1, GCLC,GCLM proteins	Han et al., 2014
Rat	SD; M; 275–300 g	Dimethyl fumarate (DMF)	• Post; 50 mg/kg; gavage; 2–3 h after MCAO until d14; twice daily	• Infarct volume (14 d) ↓	• Neurological deficits (3, 7, 14 d) ↓	• Nrf2 (total ↑, at 110 KDa) and HO1 proteins	Lin et al., 2016
Rat	SD; M; 8–10 wks; 250–300 g	Tissue kallikrein (TK)	• Post; 8.75 × 10^−3^ PNAU/kg; i.v.; immediately after reperfusion; • once	• Infarct volume (1 d) ↓	• Neurological deficits (1 d) ↓	• Nrf2 (total ↑) and HO1 proteins	Yang et al., 2017
Rat	SD; M; 57–61 d; 250–280 g	Compound 10 b	• Post; 140 mg/kg; gavage; immediately after MCAO • Once	• Infarct volume (1 d) ↓ • Brain edema (1 d) ↓	• Neurological deficits (1 d) ↓	• Nrf2 (total ↑; at 68 KDa) and HO1 proteins	Hua et al., 2015
Rat	SD; M; 260–280 g	YQ138	• Post; 10 mg/kg; i.v.; 2, 4, and 6 h after MCAO onset • Three times	• Infarct volume (1 d) ↓ • Brain edema (1 d) ↓	• Neurological deficits (1 d) ↓	• Nrf2 (total ↑) and HO1 proteins	Pang et al., 2016
Rat	SD; M; 220–240 g	Protocatechualdehyde (PCA)	• Post; 40 mg/kg; i.v.; 1 h before reperfusion • Once	• Infarct volume (1 d) ↓	• Neurological deficits (1 d) ↓	• Nrf2 (total ↑) and HO1 proteins	Guo et al., 2017
Rat	SD; M; 280–300 g	11-Keto-β-boswellic acid (KBA)	• Post; 25 mg/kg; i.p.; 1 h after reperfusion • Once	• Infarct volume (2 d) ↓	• Neurological deficits (2 d) ↓	• Nrf2 (total ↑) and HO1 proteins • Nrf2 and HO1 (IF)	Ding et al., 2015
Rat	SD; M; 230–260 g	Z-ligustilide (LIG)	• Post; 32 mg/kg; i.v.; immediately after MCAO • Once	• Infarct volume (1 d) ↓	• Neurological deficits (1 d) ↓	• Nrf2 (total ↑) protein	Peng et al., 2013
Rat	Wistar; M; 280–300 g	Dihydrocapsaicin (DHC)	• Post; 10 mg/kg; i.p.; 15 min before reperfusion • Once	• Infarct volume (1 d) ↓	• Neurological deficits (1 d) ↓	• Nrf2 (total ↑; at 68 KDa) and NQO1 proteins • SOD, GPx activity	Janyou et al., 2017
Rat	SD	Britanin	• Pre or Post; 50 mg/kg; gavage; 2 h before MCAO to 2 h after MCAO • Once	Infarct volume (1 d) ↓	• Neurological deficits (1 d) ↓	• Nrf2 (total ↑) and HO1, NQO1 proteins	Wu G. et al., 2017
Rat	SD; M; 250–300 g	4-Hydroxybenzyl alcohol (4-HBA)	• Pre; 50 mg/kg; i.p.; daily • For 3 d	• Infarct volume (1 d) ↓	• Neurological deficits (0, 1, 2 d) ↓	• Nrf2 (IF)	Yu et al., 2013
Rat	SD; M; 260–280 g	Water extract (GUW) of *Gastrodia elata* and*Uncaria rhynchophylla*	• Post; 288.6 mg/kg; gavage; daily • For 7 d	• Infarct volume (7 d) ↓	• Neurological deficits (3, 5, 7 d) ↓ • Beam-walking (3, 5, 7 d) ↓	• Nrf2 (IHC)	Xian et al., 2016
Rat	SD; M; 7–8 wks; 250–280 g	Salidroside	• Pre and Post; 30 mg/kg; i.p.; immediately prior to MCAO and immediately after reperfusion • Twice	• Infarct volume (1 d) ↓	• Neurological deficits (1 d) ↓	• Nrf2 (IHC)	Han et al., 2015

*MCAO, middle cerebral artery; pdMCAo, permanent distal middle cerebral artery occlusion; pMCAO, permanent (proximal end of) middle cerebral artery occlusion that is generated by the intraluminal suture MCAO; GCI, global cerebral ischemia; i.p., intraperitoneal; i.v., intravenous; i.c.v., intracerebroventricular; Pre, pretreatment; Post, posttreatment; the changes in brain lesion/edema and neurobehavioral deficits/Nrf2 protein expression level rf2 protein expression leveledema and neurobehavioral deficitseatmest, posttreatmeatme; IF, Immunofluorescence; KDa, kilodalton that indicates Nrf2 protein molecular weight by Western blot; the changes in brain lesion, edema, neurological deficits, and mRNA/protein expression level (↑ or ↓, increase or decrease at indicated time point; no label at indicated time point, no significant difference); h, hour; d, day; wk, week*.

The activation of the Nrf2/ARE pathway following global ischemia damage remains controversial (Table 4). This might be because of the various experimental factors like animal background, age, model, observation time points after ischemia, presence or absence of reperfusion, and site of the sample. At 24 h after GCI (2 vessel occlusion, 2VO), hippocampal Nrf2 content increased nearly 2-fold as determined by biochemical assay (Atef et al., 2018); striatal nuclear Nrf2 protein level and DNA binding activity of Nrf2 increased about 1.5-fold following reperfusion compared with the sham group; and there was notable upregulation in the detoxification and antioxidant proteins HO1, NQO1, GCLC, and GCLM (Ya et al., 2017). At 3 days after GCI (2VO), the total, cytoplasmic, and nuclear protein levels of Nrf2 were upregulated in the hippocampal CA1 region of rats although the increase in nuclear Nrf2 was not significant (Chen B. et al., 2016), a finding which is supported by another report (Lei et al., 2016). No change in HO1 protein level was detected at 3 days following brain injury (Chen B. et al., 2016). An immunofluorescence-based study showed that the Nrf2 level was slightly (but not significant) higher in the rats hippocampal CA1 area along with a decline of HO1 expression at 7 days after GCI (2VO) (Tulsulkar and Shah, 2013). In a rat GCI (4VO) model, activation of Nrf2 in the hippocampal CA1 region was examined over 3 days by four metrics including nuclear translocation of Nrf2, total Nrf2 protein level, DNA-binding of Nrf2, and induction of Nrf2 regulated proteins (Tu et al., 2015). The cytosolic but not nuclear Nrf2 protein was slightly upregulated from 12 to 72 h as evidenced by WB and immunostaining analyses, whereas the total Nrf2 protein level remained unchanged at 24 h. No change in the Nrf2 DNA binding activity was detected at 24 h and 72 h. The Nrf2-regulated antioxidant proteins HO1, NQO1, SOD2, and GPx1 were slightly altered within 72 h. These findings are supported by another report of GCI with less time of ischemia (Wang et al., 2013) that presented the upregulation of HO1 protein at 3 days. Lastly, it should be pointed out that the reports are inconclusive concerning the protein expression levels of Nrf2 with its target genes and subcellular distribution of Nrf2 following GCI. For example, hippocampal Nrf2 protein was reported to have not changed (Yang Y. et al., 2015), significantly decreased (Liu H. et al., 2015), or increased (Ashabi et al., 2015; Lee et al., 2015) following ischemia.

**Table 4:** Preclinical studies of Nrf2 in global cerebral ischemia models of mice and rats.

**Model**	**Species**	**Genetic Background; Sex; Age or Weight**	**Treatment**	**Dosage/Administration route**	**Brain lesion/Edema**	**Neurobehavioral deficits**	**Nrf2 Mechanism (*in vivo*)**	**References**
**Findings supporting the role of Nrf2 pathway (with Nrf2**^**−/−**^ **mice)NA**								
**Findings supporting the role of Nrf2 pathway (without Nrf2**^**−/−**^ **mice, indicated by Nrf2 protein nuclear translocation)**								
*2VO-20 min*	Mouse	C57BL/6; M; 8 wks; 20-22 g	5-Hydroxymethyl-2-furfural (5-HMF)	• Pre and post;12 mg/kg; i.p.; 30 min before AND 5 min after the onset of reperfusion • Twice	• Neuronal injury (striatum; via cresyl violet, TUNEL; 1 d) • Brain edema (both hemispheres; 1 d) ↓	• Neurological deficits (24 h) ↓ • Locomotor activity (24 h) ↓ • Inclined beam walking (24 h) ↓	• Nrf2 (nuclear ↑) and HO1, GCLC, GCLM, NQO1 proteins • DNA binding activity of Nrf2	Ya et al., 2017
*2VO-20 min*	Rat	SD; M; 250–300 g	Rifampicin	• Post; 20 mg/kg; i.p.; 30 min after onset of reperfusion; daily; • For 7 d	• Neuronal death (CA1; via HE, TUNEL; 7 d) ↓	• Morris water maze (8, 9, 10 d) ↓	• Nrf2 (nuclear ↑, cytoplasmic ↓, total) and HO1 proteins	Chen B. et al., 2016
*4VO-10 min*	Rat	SD; 320–360 g	Sevoflurane	• Post; inhalation of 2% sevoflurane for 10 min; inhalant; twice after ischemia	• Neuronal necrosis (CA1; via HE, TUNEL; 1, 7 d) ↓	• Neurological deficits (1, 7 d) L;	• Nrf2 (nuclear ↑) and HO1 proteins	Lee et al., 2015
*4VO-10 min*	Rat	SD; M; 250–300 g	Genistein	• Post; 1 mg/kg; i.c.v.; 5 min after onset of reperfusion • Once	• Neuronal death (CA1; via NeuN, TUNEL; 5 d) ↓	• Morris water maze (7, 8, 9 d) ↓	• Nrf2 (nuclear ↑; cytoplasmic ↓) protein • DNA Binding Activity of Nrf2 • HO1 protein (WB, staining)	Wang et al., 2013
*4VO-15 min*	Rat	SD; M; 250–300 g	DEETGE-CAL-Tat peptide	• Pre; 50 μg; i.c.v.; 30 min before ischemia; once • Post; 240 μg/d; subcutaneously; 1 d after ischemia until d9; daily	• For pretreatment: • Neuronal Injury (CA1; via NeuN, TUNEL; 7 d) ↓ For posttreatment: • Neuronal Injury (CA1; via NeuN, TUNEL; 9 d) ↓	• Morris water maze (7, 8, 9 d) ↓	• Nrf2 (nuclear ↑, cytoplasmic ↓) protein • Nrf2 DNA binding HO1, NQO1, GPx1, and SOD2	Tu et al., 2015
*2VO-20 min*	Mouse	C57BL/6; M; 12 wks; 20–24 g	Lycopene	• Pre; 20 mg/kg; i.p.; daily • For 7 d	• Neuronal degeneration (CA1; via HE, TUNEL; 3 d) ↓	• Neurological deficits (1, 2, 3 d) ↓	• Nrf2 (nuclear ↑, total ↑) and HO1 proteins	Lei et al., 2016
**Other findings involving the role of Nrf2 pathway**								
*2VO-permanent*	Rat	SD; M; 180–250 g	Sodium butyrate (SB)	• Post; 840 mg/kg; i.p.; 29–56 d after ischemia; daily; • For 28 d	• Immunoreactivity of neuronal/ synaptic proteins (HC; via NeuN; 56 d)	• Morris water maze (50-56 d) ↓ • Novel object recognition (49–56 d)	• Nrf2 (total ↑) protein Nrf2 • down-stream genes mRNA levels (GCLc, HO1, NQO1 and GCLm)	Liu H. et al., 2015
*2VO-permanent*	Rat	Wistar; M; 7 wks; 220–250 g	Environment enrichment (EE)	• Post; 6 h/d; daily • For 28 d	• Oxidative neuronal damage (CA1, CA2, CA3; via 4-HNE; 56 d) ↓	• Morris water maze (3-5 d) ↓	• Nrf2 (total ↑) protein	Yang Y. et al., 2015
*4VO-30 min*	Rat	Wistar; M; 6 mo; 270–290 g	Metformin (MF)	• Pre; 200 mg/kg; gavage; daily • For 14 d	• NA	• NA	• Nrf2 (total ↑; at 68 KDa) and HO1 proteins	Ashabi et al., 2015
*2VO-45 min*	Rat	Wistar; M; 250–270 g	N6-cyclohexyl adenosine (CHA)	• Post; 6.25 nM in 1 μl; unilateral intrahippocampal injection; immediately after onset of reperfusion; • Once	• Neuronal degeneration (hippocampus; via HE; 1 d)	• Morris water maze (1 d) ↓ • Rotarod (1 d) r • Open field (1 d) ↓	• Nrf2 content (Elisa)	Atef et al., 2018
*2VO-8 min*	Mouse	C57BL/6; M; 8–10 wks; 20–25 g	Ginkgo biloba/EGb 761® (EGb 761)	• Pre; 100 mg/kg; gavage; daily; • For 7 d	• Neuronal injury (CA1; TUNEL; 7 d) ↓	• NA	• Nrf2 and HO1 (IF)	Tulsulkar and Shah, 2013

*MCAO, middle cerebral artery; pdMCAO, permanent distal middle cerebral artery occlusion; pMCAO, permanent (proximal end of) middle cerebral artery occlusion that is generated by the intraluminal suture MCAO; GCI, global cerebral ischemia; i.p., intraperitoneal; i.v., intravenous; i.c.v., intracerebroventricular; Pre, pretreatment; Post, posttreatment; the changes in brain lesion/edema and neurobehavioral deficits/Nrf2 protein expression level rf2 protein expression leveledema and neurobehavioral deficitsntt is generated by the; IF, Immunofluorescence; KDa, kilodalton that indicates Nrf2 protein molecular weight by Western blot; the changes in brain lesion, edema, neurological deficits, and mRNA/protein expression level (↑ or ↓, increase or decrease at indicated time point; no label at indicated time point, no significant difference); h, hour; d, day; wk, week*.

## Functional Benefit of NRF2 In Cerebral Ischemia—*in vivo* Evidence From NRF2^−/−^ Mice

Multiple lines of evidence demonstrate the beneficial contribution of Nrf2 in various pathological conditions, and most come from studies using Nrf2 knockout (Nrf2^−/−^) or knockdown animals. Reports showed that Nrf2^−/−^ mice do not exhibit any overt abnormal phenotype regarding size, body weight, food intake, mobility, fertility, or other characteristics at baseline (Itoh et al., 1997; Wang et al., 2007; Wang Y. C. et al., 2011; Wang B. et al., 2012; Leonardo et al., 2013, 2015; Doré, 2015; Liu et al., 2018). In addition, Nrf2^−/−^ mice exhibit similar cerebrovascular architecture in anastomosis, vascular physiology, and blood pH compared to their WT counterparts (Leonardo et al., 2015). Basal GST and NQO1 activities, but not GSH content, modestly but significantly decreased in multiple brain regions of Nrf2^−/−^ mice compared to their Nrf2^+/+^ littermates, suggesting that the constitutive synthesis of brain GSH does not rely on Nrf2 function, at least in young adult mice (Shih et al., 2005).

However, in response to the various ischemic insults, it is becoming clear that Nrf2 plays a critical role in protection against ischemic brain damage (Tables 1–3). Indeed, older and recent studies with different cerebral ischemia models have revealed the functional benefit of Nrf2 on infarct volume, brain edema, and neurobehavioral deficits after ischemia. Following permanent ischemia injury without reperfusion, Nrf2 deficiency exacerbated the acute development of brain lesions, revealed by severe infarct volume at 3 days but not at 1 day after pdMCAO, and these mice exhibited relatively poor sensorimotor function over 28 days (Liu et al., 2018). At 2 days after pdMCAO, Nrf2^−/−^ mice had significantly larger cortical infarct volume and more severe neurological deficits than WT controls (Wang B. et al., 2012). Another group showed that Nrf2 deficiency slightly (but not significantly) enlarged the cortical infarct size at 1 day and sustained considerably more damage at 7 day after pdMCAO (*P* < 0.01), which further supports the findings above (Shih et al., 2005). Moreover, it is well-known that reperfusion after a long period of ischemia can exacerbate brain damage. Following cerebral ischemia injury with reperfusion, several studies showed that Nrf2^−/−^ mice exhibited aggravated acute brain damage in infarct volume and neurological deficits scores at 1 day after tMCAO with 1 h (Li et al., 2013; Yao et al., 2016; Zhang et al., 2017), 1.5 h (Shah et al., 2007), and 2 h (Shi et al., 2015) of ischemia. In these studies, no significant difference in blood pH, PaO_2_, PaCO_2_, or cerebral blood fluid (CBF) was observed before, during, or after ischemia-reperfusion between WT and Nrf2^−/−^ at indicated time points as monitored by laser-Doppler flowmetry (Shah et al., 2010). In contrast, another contradictory report showed that no significant difference in infarct volume was observed between Nrf2^−/−^ and WT mice 2 days after 1 h tMCAO (Narayanan et al., 2015). This discrepancy might be related to the different genetic background or age of mice or the experimental setting.

Nrf2 disruption depresses the capacity of the cellular antioxidant system and diminishes upregulation of its target cytoprotective proteins in response to ischemia (Tables 1–3). A study showed that, along with severe ischemic brain damage 3 days after pdMCAO, Nrf2 deficiency destroyed the ischemia-induced increase of antioxidant/detoxification proteins NQO1, HO1, SOD2, and Gpx1 (Liu et al., 2018). Oxidative stress and inflammation plays a key role in the pathological process of ischemia. Excessive production of ROS and reactive nitrogen species (RNS) contributes to the activation of inflammatory processes, further aggravating brain injury and functional deficits during or after stroke. Toll-like receptor 4 (TLR 4) initiates inflammatory processes and induces the expression of inflammatory elements IL-6, TNFα, and IL-1β through activation and nuclear translocation of NF-κB in cerebral ischemia/reperfusion injury (Arslan et al., 2010; Wang Y. C. et al., 2011). At 24 h after 1 h tMCAO, Nrf2 knockout upregulated the TLR4 and NF-κB proteins mediated by the inflammatory response in the ischemic cortex (Li et al., 2013).

Astrocytes interact closely with neurons to provide structural and functional support at multiple levels, including ion and water homeostasis, chemical signal transmission, blood flow regulation, immune and oxidative stress defense, and supply of metabolites (Liu et al., 2017). Given that the ARE-regulated genes are preferentially activated in glial cells, which have more effective detoxification and antioxidant capacities than neurons (Vargas and Johnson, 2009; Gan et al., 2012; Haskew-Layton et al., 2013), Nrf2 in glia cells can protect neurons from a wide array of insults like stroke (Vargas and Johnson, 2009). In response to ischemic insults, astrocytes undergo structural and functional changes, and interact with other glial cells to defend neurons (Magaki et al., 2018). Indeed, in the ischemic cortex, Nrf2 deficiency exacerbated reactive astrogliosis, dysfunction in glutamate metabolism, and water permeability within 3 days after pdMCAO. The spatiotemporal pattern of reactive astrogliosis correlated well with acute ischemic damage progression. Interestingly, the microglia activation was affected considerably under Nrf2 absence.

## NRF2 Targeted Intervention In Ischemic Stroke—Advancements From Experimental Ischemic Stroke Model

Given the fundamental role of Nrf2 in redox homeostasis, many studies have presented the unique contribution of Nrf2 in neuroprotection against various diseases (Ma, 2013; Kumar et al., 2014; Sun et al., 2017; Cuadrado et al., 2018). For ischemic stroke, Nrf2/ARE activation is expected to trigger a cytoprotective response counteracting deleterious ischemic events. The beneficial effects of some candidate Nrf2 inducers were directly proved by the findings in Nrf2^−/−^ mice. The typical findings were listed below (just to name a few), and more are also depicted in Tables 1–4.

### Dimethyl Fumarate and Monomethyl Fumarate

Dimethyl fumarate (DMF) and its primary metabolite monomethyl fumarate (MMF) are two typical Nrf2 inducers. Several studies support their neuroprotective efficacy against ischemic brain injury through the activation of the Nrf2/HO1 pathway. DMF and MMF dramatically reduced infarct volume, brain edema, and neurological deficits over 7 days after tMCAO, along with the suppressed acute glial activation (Yao et al., 2016). Importantly, such evident protection was abolished in Nrf2^−/−^ mice, indicating that the Nrf2 pathway is required for the benefit of DMF and MMF. During the late phase (7–14 d), DMF also acted as a potent immunomodulator, reducing the infiltration of neutrophils and T cells and the number of activated microglia and macrophages in the infarct region (Lin et al., 2016). Interestingly, DMF also attenuated brain edema formation during ischemia and stabilized the blood brain barrier (BBB) by preventing disruption of interendothelial tight junctions (Kunze et al., 2015). DMF protected against acute brain damage in edema volume and sensorimotor deficits after pdMCAO through anti-inflammatory cytokines (IL-10 and IL-12p70), further supporting the neuroprotective role of MMF (Clausen et al., 2017).

### Sulforaphane

Sulforaphane (SFN), a typical Nrf2 activator, is a naturally occurring isothiocyanate found in cruciferous vegetables. Upregulation of Nrf2 by sulforaphane pretreatment was associated with increased HO1 expression in perivascular astrocytes in the peri-infarct regions and cerebral endothelium of the infarct core. BBB disruption, lesion progression, and neurological deficits were reduced after tMCAO. This indicates that the Nrf2 defense pathway in the cerebral microvasculature provides a novel therapeutic approach for preventing BBB breakdown and neurological dysfunction in stroke victims (Alfieri et al., 2013). There is a contradictory finding that sulforaphane treatment increased the overall mRNA levels of transcription of Nrf2, Hmox1, GCLC and GSTA4 but failed to reduce the infarct volume and motor deficits in a photothrombotic mouse model (Porritt et al., 2012).

### Tert-Butylhydroquinone

Tert-butylhydroquinone (tBHQ) is a Nrf2 inducer widely used as a food additive. Nrf2 activation by tBHQ pretreatment reduced cortical damage and sensorimotor deficits from 24 h up to even 1 month after pdMCAO. Interestingly, larger infarcts were observed in Nrf2^−/−^ mice 7 d after stroke, but not 24 h after stroke (Shih et al., 2005). This Nrf2-specific action of tBHQ *in vivo* was also validated in another study with tMCAO (Shah et al., 2007). A recent study showed that Nrf2 upregulation by tBHQ significantly reduced the expression of cytoplasmic thioredoxin interacting protein, nod-like receptor protein 3 (NLRP3) inflammasome, and downstream factors caspase-1, IL-1β, and IL-18, all of which contribute to the Nrf2-mediated neuroprotection against ischemic outcomes after tMCAO (Hou et al., 2018). Interestingly, there is a contradictory report that tBHQ-exacerbated stroke damage (Sun et al., 2016), revealed by increased post-stroke mortality and worsened outcomes after pMCAO.

### MiR-93 Antagomir

MicroRNAs (miRNAs) play vital roles in regulating neuronal survival during cerebral ischemia/reperfusion injury. MiR-93, a direct negative modulator of Nrf2 expression at the transcriptomic level, serves as a potential therapeutic target for acute ischemic stroke. MiR-93 levels in the ischemic cortex of mice increased at 24 h and 48 h after tMCAO. MiR-93 antagomir treatment reduced infarction volume, neural apoptosis and neurological deficits through the Nrf2/HO1 antioxidant pathway (Wang et al., 2016).

### Korean Red Ginseng

Korean Red Ginseng (Ginseng) is one of the most widely used herbal medicines with reported antioxidant and anti-inflammatory properties, displaying promising potential in neuroprotection. Ginseng pretreatment ameliorated short- and long-term sensorimotor deficits over 28 days, prevented the acute enlargement of lesion volume, attenuated reactive astroglial progression but not microglial activation, and enhanced the induction of Nrf2 target antioxidant proteins after pdMCAO in WT mice, an effect that was abolished in Nrf2^−/−^ mice (Liu et al., 2018). Nrf2-dependent spatiotemporal reactive astrogliosis correlated well with acute ischemic damage progression. In contrast, Nrf2 deficiency exacerbated the ischemic conditions. This supported the conclusion that Ginseng pretreatment protects against acute sensorimotor deficits and promotes its long-term recovery after pdMCAO, at least partly, through Nrf2 activation. Attenuated reactive astrogliosis contributes to the Nrf2-depedent neuroprotection.

### (–)-Epicatechin

The (**–**)-Epicatechin (EC) is especially abundant in cocoa, dark chocolate, and green tea, and it boosts antioxidant activity while supporting vascular function. EC-treated ischemic WT mice displayed a reduction of forelimb motor coordination impairments associated with reduced anatomical injury and microglia/macrophage activation in pMCAO and tMCAO models of adult WT mice, impairments which were abolished in tissues and neurons from Nrf2^−/−^ mice (Shah et al., 2010). Interestingly, this neuroprotection was observed in 12 month-old WT and Nrf2^−/−^ mice, indicating that age influences Nrf2 function (Leonardo et al., 2013, 2015).

### Resveratrol

Resveratrol is a natural polyphenolic compound that is found in some dietary sources such as grapes, plums, and red wine. *Trans*-resveratrol pretreatment had neuroprotective effects on ischemic brain damage after tMCAO. This neuroprotective effect is likely exerted by upregulated expression of Nrf2, HO1 and SOD that ameliorates oxidative damage (Ren et al., 2011). Meanwhile, resveratrol preconditioning-mediated neuroprotection is reduced after tMCAO (Narayanan et al., 2015). WT and Nrf2^−/−^ cortical mitochondria exhibited increased uncoupling and ROS production after resveratrol pre-treatments. Nrf2^−/−^ astrocytes exhibited decreased mitochondrial antioxidant expression and were unable to upregulate cellular antioxidants.

### Carbon Monoxide

Carbon monoxide (CO) is a gaseous second messenger produced when heme oxygenase enzymes catabolize heme. At low doses (Otterbein et al., 1999; Koehler and Traystman, 2002; Leffler et al., 2011), despite being traditionally viewed as a toxic agent (Cheng et al., 2012), CO displayed sustained neuroprotective efficacy against brain damage and progressive functional deficits via the Nrf2 pathway in tMCAO and pdMCAO models (Meffert et al., 1994; Maines, 1996; Alkadhi et al., 2001). Brain lesions in mice exposed to CO were 29.6 ± 12.6% smaller at 7 days after pdMCAO. Additionally, 18-h CO treatment led to Nrf2 dissociation from Keap1, nuclear translocation, increased activity of Nrf2 binding to the ARE sequence of the HO1 gene, and elevated HO1 expression from 6 to 48 h after CO exposure, the neuroprotection of which was abolished in Nrf2^−/−^ mice (Wang B. et al., 2011).

### Tetramethylpyrazine

Tetramethylpyrazine (TMP) is one of the main components of Ligusticum wallichii Franchat (Chuan Xiong) that has been used to treat neurovascular and cardiovascular diseases including stroke in traditional Chinese medicine. TMP has been found to protect against pdMCAO injury and reduce inflammation and ischemia-induced neutrophils through Nrf2/HO1 activation (Kao et al., 2013; Chang et al., 2015).

### Curcumin

Curcumin is a phenolic pigment extracted from the rhizome of *Curuma longa* Linn, which has been widely used as medicine in many Asian countries. It has strong neuro-protective effects on neurological injuries, with virtually no toxicity, even at a high dose; though, its absorption-bioavailability is the main issue, and few groups are actively looking to test innovative formulation to circumvent this serious limitation. Curcumin significantly reduced infarct size, brain edema, neurological dysfunction, and oxidative stress levels in tMCAO rats, and this neuroprotective effect involves the Akt/Nrf2 and NF-κB pathways (Wu J. et al., 2013; Li et al., 2016).

Besides the candidate Nrf2 inducers above, many natural products, synthetic compounds and clinical drugs, such as ginkgo biloba (Tulsulkar and Shah, 2013), paeonol (Zhao et al., 2014), isoquercetin (Chen M. et al., 2017), tanshinone IIA (Cai et al., 2017), ursolic acid (Li et al., 2013), isorhamnetin (Zhao et al., 2016), docosahexaenoic acid (Chang et al., 2013), HP-1c (Wang et al., 2018), gastrodin (Peng et al., 2015), trichostatin A (Wang B. et al., 2012), bicyclol (Zhang J. et al., 2014), recombinant human erythropoietin (Meng et al., 2014), omega-3 fatty acids (Zhang M. et al., 2014), Tocovid (Shang et al., 2018), estradiol (Li et al., 2017), and more, have also been shown to benefit ischemic brain injury through mechanisms involving Nrf2.

## Pitfalls and Considerations of Current NRF2 Experimental Ischemic Stroke Studies

Although most reports support the beneficial effect of Nrf2 activation by a multitude of variety of inducers against ischemic brain damage, many findings regarding the role of Nrf2 during ischemia or ischemic-reperfusion injury are contradictory. This could be attributed to a number of inconsistencies across studies, as briefly overview here. (1) Nrf2 antibody: Getting potent, selective and highly specific antibodies against the various species of Nrf2 has been an ongoing serious issue. Furthermore, what is the correct molecular weight of Nrf2 protein? The biologically relevant molecular weight of Nrf2 protein has been proven to be ~95–110 KDa, but not ~55–65 KDa, based on its 2-kb open reading frame or its tertiary structure (Lau et al., 2013; Kemmerer et al., 2015). Many reports presented the expression level of Nrf2 protein by Western blots at apparent different (or unspecified) molecular weights, which might be mainly responsible for the contradictory results among research groups, the lack of good negative controls to be run in parallel should be documented. Additionally, this issue may also contribute to the discrepancy regarding findings of Nrf2 in subcellular distribution and cell-type expression under normal and inducible conditions. (2) Questions remain as to which cell types would be most responsible for the neuroprotection associate with Nrf2. (3) Some reports also questioned the potential issues in the Nrf2 knockout lines and strains. (4) Furthermore, while many of these reported cytoprotective enzymes that are induced by Nrf2 activation, there are activate debates as to whether traditional freely available substrates are sufficiently high to explain the resulting beneficial properties of these metabolites/end-products. (5) While many of these compounds are assumed to be the bioactive entity, there are well-recognized issues on such drugs to be bioavailable and cross the blood brain barrier to then reach the target at a concentration that would be biologically relevant; thus, there is the possibility that indirect pathways are stimulated that would then indirectly activate the Nrf2 pathway. (6) Several of the studies have tested preventive medicine when give before ischemia while others have tested the therapeutic potential of the given drug concoction; though, one should not extrapolate conclusion of the universal protection until it is rigorously tested and independently validated. (7) Nrf2/ARE pathway: Is the Nrf2/ARE pathway activated or not? To validate that Nrf2 is activated, many reports based their conclusions on selected protein level measurements of total Nrf2 with or without its target markers like HO1 by WB. Based on the current understanding of Nrf2 activation, nucleus translocation of Nrf2 is essential to its activation, which is revealed by nuclear Nrf2 protein levels. Evidence that targets distinct components of the Nrf2/ARE pathway cascade will be expected. In addition, the application of pharmacological or genetic blockage of Nrf2 will be helpful to demonstrate whether Nrf2/ARE pathway activation is specific to the neuroprotection of the candidate Nrf2 inducer. (8) Ischemic brain tissue: Which part of the ischemic cerebral hemisphere was used for measurement? Despite variable ischemic damage to different brain regions (or together with reperfusion), various ischemic brain tissue like the cortex, ischemic region, and cerebral hemisphere was used for measurement, possibly leading to inconsistent results. These tissue samples such as the infarct core, peri-infarct, and healthy brain regions, could “dilute” and even mask the changes of markers interest during cerebral ischemia. The apparent alternation of Nrf2 expression happens inside the peri-infarct area. Accordingly, tissue from the same site of the peri-infarct area appears to be appropriate for the subsequent measurement that leads to the final conclusion. (9) Histological and neurobiological outcomes: Which marker and which time points are expected to have the functional benefit of a potent Nrf2 inducer? Most reports only selected infarct volume or neurological deficit score in the acute ischemic stage (1–3 days) without tracking long-term efficacy. As we know, ischemic insult induces severe motor, sensory, emotional, and cognitive deficits (Ferro et al., 2016), and long-term functional recovery is considered to be the goal of stroke intervention. More accurate and long-term histological and functional evaluations are expected for future studies. (10) Nrf2 activators: As mentioned above, a number of Nrf2 activators have been identified that appears to upregulate Nrf2/ARE pathway. However, thus far, only limited compounds including sulforaphane, CDDO-methyl ester, and DMF have consistently indicated sufficient pharmacokinetic and pharmacodynamic properties to act in brain (Lastres-Becker et al., 2016; Yamamoto et al., 2018). Future rigorous studies are needed to provide the necessary information concerning the clinical application prospect of Nrf2 targeted intervention.

This review focusses on Nrf2 activity in the context of ischemic stroke, though, over- or prolonged Nrf2 activation could potentially be problematic. For example, sustained activation of Nrf2 in drosophila would shorten its life expectancy (Tsakiri et al., 2013), and it would also promote the malignant transformation of human cells (Yang X. et al., 2015). It has also been reported that mutations in Nrf2 and Keap1 would promote cancer cell survival (Shibata et al., 2008); furthermore, many cancer cells would overexpress Nrf2 and this would be associated with cell resistance to cancer therapies (Taguchi and Yamamoto, 2017).

## Conclusive Remarks And Perspective

Emerging evidence demonstrates that the Nrf2 network plays a crucial role in cellular adaption by controlling a wide range of cytoprotective proteins, counteracting distinct endogenous and exogenous insults while providing a promising optimal therapeutic target against various diseases from cancer to brain disorders. By using various ischemic stroke rodent models, recent preclinical studies provide direct *in vivo* evidence revealing the contribution of the Nrf2 pathway in ischemic stroke pathogenesis and neuroprotection. This review highlighted the promising potential of interventions targeting Nrf2, implying that the moderate activation of Nrf2 favors the attenuation of brain damage and long-term recovery from cerebral ischemia. Because the investigations of Nrf2 in stroke are still in the initial stages, future research is expected to elucidate the natural properties of Nrf2 in stroke leading to the development of novel drugs that target Nrf2.

## Author Contributions

LL and SD conceived the study and designed the databases analysis. LL prepared the manuscript with input from SD. LL and LML searched databases, collected data, performed analyses, and prepared the tables and figure. All authors reviewed, discussed and approved the final manuscript.

### Conflict of Interest Statement

The authors declare that the research was conducted in the absence of any commercial or financial relationships that could be construed as a potential conflict of interest.
